# Efficient and flexible simulation-based sample size determination for clinical trials with multiple design parameters

**DOI:** 10.1177/0962280220975790

**Published:** 2020-12-02

**Authors:** Duncan T Wilson, Richard Hooper, Julia Brown, Amanda J Farrin, Rebecca EA Walwyn

**Affiliations:** 1 Leeds Institute of Clinical Trials Research, University of Leeds, Leeds, UK; 2 Centre for Primary Care & Public Health, Queen Mary University of London, London, UK

**Keywords:** Clinical trials, simulation, sample size, power, Gaussian process, global optimisation

## Abstract

Simulation offers a simple and flexible way to estimate the power of a clinical trial when analytic formulae are not available. The computational burden of using simulation has, however, restricted its application to only the simplest of sample size determination problems, often minimising a single parameter (the overall sample size) subject to power being above a target level. We describe a general framework for solving simulation-based sample size determination problems with several design parameters over which to optimise and several conflicting criteria to be minimised. The method is based on an established global optimisation algorithm widely used in the design and analysis of computer experiments, using a non-parametric regression model as an approximation of the true underlying power function. The method is flexible, can be used for almost any problem for which power can be estimated using simulation, and can be implemented using existing statistical software packages. We illustrate its application to a sample size determination problem involving complex clustering structures, two primary endpoints and small sample considerations.

## 1. Introduction

The sample size of a clinical trial is typically chosen with respect to its power, which is the probability of a statistically significant result conditional on the parameter of interest being equal to the minimal clinically important difference (MCID).^1^ Since power will generally increase with sample size, a nominal power threshold (often 80% or 90%) is set and the smallest sample size which satisfies this is selected. For many sample size determination (SSD) problems, power can be calculated using a simple mathematical formula and the optimisation problem can be solved in a timely manner. When complexity in the trial design or the method of analysis mean such formulae are not readily available, we can estimate power using a Monte Carlo (MC) approximation.^2,3^ To do so, we simply simulate several hypothetical sets of trial data under the alternative hypothesis, analyse each of these, and calculate the proportion of analyses which reject the null hypothesis. The simplicity and flexibility of the simulation method has seen it used for a variety of statistical models and study designs, including problems involving hierarchical models,^4,5^ proportional hazards models,^6^ logistic regression models,^7^ individual patient data meta-analyses,^8^ patient enrolment models,^9^ stepped wedge designs^10,11^ and cluster randomised crossover designs.^12^ Although calculating MC estimates of power can be computationally demanding, these SSD problems remain feasible because, as optimisation problems, they are quite simple. In particular, optimisation takes place over a single parameter (the sample size), subject to a single constraint (power), and with respect to a single objective to be minimised (the sample size again).

SSD problems, particularly those found in trials of complex interventions, are not always this simple.^13^ There may be several dimensions to the trial’s sample size or, more generally, several quantitative parameters, which must be specified at the design stage and which influence the power of the trial. We will refer to these as *design parameters*. Several design parameters are common in, for example, trials with multilevel structures such as cluster randomised trials, where both the number of clusters and the number of participants in each cluster must be specified. Increasing the number of design parameters complicates the SSD problem by increasing the number of possible solutions to search. A second complication arises when there is more than one criterion we are interested in minimising, subject to the nominal power constraint. A cluster randomised trial will often have this property, as we would like to minimise both the total number of participants and the number of clusters. Given multiple conflicting objectives, there is no single ‘optimum’ solution but rather a range of solutions which offer different degrees of trade-off between the objectives. Seeking a set of good solutions, rather than a single optimum, further adds to the difficulty of the SSD problem.

Simple, simulation-based SSD problems can generally be solved in a feasible time with an exhaustive search,^14,15^though a bisection search, as proposed by Williams et al.^16^ and refined by Jung,^17^ may be more efficient. A more sophisticated algorithm was implemented in the SimSam package,^5^ written for Stata (Stata Corporation, College Station, TX, USA). Although there is no such general framework for solving complex SSD problems, a number of methods have been proposed for specific applications. In some cases, these methods impose restrictions which reduce the complex SSD problem to a simple one in a single dimension. For example, approaches to simulation-based SSD for stepped wedge trials have focused on choosing one design parameter (such as the number of clusters randomised to each sequence) while keeping others (the number of participants per cluster and the number and arrangement of sequences) fixed.^10,11^ In other applications, including meta-analysis,^18^ work has focussed on estimating power through simulation but has left the associated complex SSD problem unspecified. The complexity of SSD in multilevel designs has been addressed to some extent in the MLPowSim package,^15^ although this is limited to performing a simple grid search over two design variables (the number of clusters, and the cluster size). Adaptive designs are another area where simulation is often used to estimate operating characteristics, and where the SSD problem is complex due to the large number of design parameters needed to describe stopping rules. Where these problems have been addressed, optimisation has remained feasible through some special structure of the problem being identified and exploited. For example, optimal multi-arm multi-stage designs can be found when the primary endpoint is normally distributed, since one can draw a single large sample from a multivariate normal distribution prior to optimisation and use transformations of these samples over the course of the search.^19^ Together with an implementation in C++, this leads to feasible computation times.

In theory, a general approach to solving any complex SSD problem would be through using benchmark multi-objective optimisation algorithms such as NSGA-II,^20^ robust implementations of which are freely available in statistical software such as R.^21^ However, these so-called ‘greedy’ algorithms typically assume that evaluating any proposed solution to the problem is a very fast process, and consequently evaluate many thousands of solutions during the search. If these algorithms were applied to problems where evaluating solutions required computing an MC estimate of power, they would take an infeasibly long time to converge. Thus, if we are to extend simulation-based trial design to complex SSD problems, we require a more general framework employing more efficient optimisation algorithms.

Outwith the context of clinical trial design, a great deal of research has addressed optimisation problems where the evaluation of a solution is a computationally demanding, or *expensive*, operation.^22^,^23^ One approach addresses the problem by substituting the expensive function with a mathematical approximation known as a *surrogate model*. The surrogate model is then used to make predictions about the true function for different values of design parameters, with these predictions informing which point should be evaluated next. The information obtained from this evaluation is used to update the surrogate model, thus improving the predictions available at the next iteration. One class of surrogate model is Gaussian process (GP) regression. Also known as Kriging and having its roots in geostatistics,^24^ GP models are spatial interpolators that are computationally tractable^25^ and can be fitted using robust and freely available software.^26^ A GP surrogate model provides not only a prediction of the true function value at any point, but also a measure of uncertainty in this prediction. This property is exploited by the benchmark efficient global optimisation (EGO) algorithm,^27^ allowing the next point in the search to be chosen in a way that formally balances the potential benefits of *exploitation* (searching around areas already known to be promising) and *exploration* (searching in areas of high uncertainty). Although EGO was originally proposed for unconstrained optimisation of expensive objective functions with deterministic output, various proposals have extended it to incorporate the expensive constraints,^28^ multiple objectives^29^ and stochastic outputs^30^ that feature in complex SSD problems.

In this paper we will explore how GP regression models and a variant of the EGO algorithm can be used to solve complex SSD problems. We will describe a general method which can be applied to almost any SSD problem, providing the user can write a programme which simulates the data generating process and analysis of the trial at any given set of parameter values and for a given choice of sample size, returning a binary indicator denoting rejection or otherwise of the null hypothesis. By allowing user-written simulation programmes, we aim to provide a flexible method which can be applied to a wide range of trial design problems. This approach will also facilitate SSD for novel designs developed in the future and which cannot be anticipated now.

The remainder of the paper is structured as follows. A motivating example is described in section 2. In section 3, we provide the necessary background and notation regarding Monte Carlo estimation and multi-objective optimisation. In section 4, we describe Gaussian process regression, the efficient global optimisation algorithm, and a framework for its application to sample size determination. We return to the examples in section 5, illustrating how the method can be applied in practice, where we also conduct a simulation study. We conclude with a discussion of the implications and limitations of the proposed approach in section 6.

## 2. Motivating example – PACE

‘Pacing, graded activity and cognitive behaviour therapy; a randomised evaluation’ (PACE)^31^,^32^ was a randomised controlled trial comparing adaptive pacing therapy, cognitive behavioural therapy, graded exercise therapy and specialist medical care as secondary care treatments for patients with chronic fatigue syndrome, with respect to two potentially correlated continuous primary endpoints (fatigue and disability). Participants were randomised to receive one of these four treatments, and we will henceforth refer to these randomised groups as ‘arms’ of the trial. The data had a complex multilevel structure, with three of the four arms including a therapy provided by different therapists (partially nested structure) and all arms including medical care from the same doctors (crossed structure), leading to the potential for treatment-related clustering. Here, we consider how we might have designed a pilot trial prior to the definitive PACE trial to provide a preliminary test of the potential efficacy of adaptive pacing therapy (APT) in comparison to specialist medical care (SMC).

**Figure 1.**
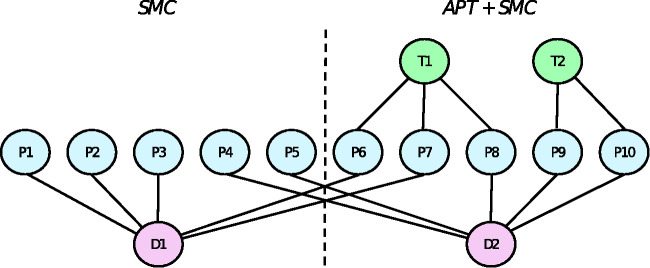
Multilevel structure of the SMC and APT arms of our example, where therapists (T) are partially nested within interventions, doctors (D) are crossed with interventions, and patients (P) are cross-classified with therapists and doctors in the intervention arm and nested within doctors in the control arm.

We assume the pilot trial would have the same complex multilevel data structure as the main trial, where therapists are partially nested within interventions, doctors are crossed with interventions, and patients are cross-classified with therapists and doctors in the intervention arm and nested within doctors in the control arm.^33^ This structure is illustrated in Figure 1. As in the original PACE trial, the primary analysis will fit a mixed effect model for each primary endpoint and use likelihood ratio tests to obtain a *p*-value in each case. By fitting separate models for each endpoint, as opposed to a joint model, misspecification will be a potential problem if there are correlations between the endpoints at the participant level, or between the random effects of therapists and doctors. Such model misspecification has been noted as a clear motivation for simulation-based power calculations,^3^ which will allow an estimate of the true power, accounting for the effect of this misspecification, to be obtained.

The results of the trial will be considered positive if either of the analyses show a statistically significant difference, leading to an inflated type I error rate under the null hypothesis of no effect on each endpoint.^34^ This inflation, together with the small sample context, motivates the inclusion of the nominal type I error rate *a* as a design parameter in this problem. Together with *a*, we have four further design parameters: the total sample size in the APT arm, denoted *n*_1_; the number of APT therapists, *k*; the allocation ratio relating the total number of participants in each arm, $r=n_{0}/n_{1}$ ; and the number of doctors, *j*. Note that we assume we can control the numbers of participants allocated to therapists and to doctors, and so can maximise efficiency by balancing cluster sizes. The three objectives to be simultaneously minimised are the total number of participants, the number of therapists, and the number of doctors. Finally, we have the two constraints of ensuring the actual type I error rate is no more than 0.2 (one-sided), and the power is at least 90%. As both type I error rate and power do not have analytical forms in this context, both constraints must be estimated using simulation.

## 3. Background

### 3.1. Monte Carlo estimation

Monte Carlo methods can be used to numerically approximate expectations E[f(Z)] of real valued functions *f*(*Z*) with respect to the probability distribution of *Z*. Given a sample of *N* independent copies of *Z*, denoted *z_i_*, i=1,…,N , the MC estimate is

(1) $$E[f(Z)]\approx \frac{1}{N}\sum_{i=1}^{N}f(z_{i})$$

The estimate is unbiased for all *N* and has variance equal to

(2) $$\omega^{2}=\mathrm{Var}\left[\frac{1}{N}\sum_{i=1}^{N}f(z_{i})\right]=\frac{1}{N}Var\left[f(z_{i})\right]$$

The standard error of the MC estimate will therefore reduce at a rate of $1/\sqrt{N}$ as we increase *N*. When *N* is large we can consider an MC estimate to be the true expectation plus a normally distributed error term with 0 mean and variance $\omega^{2}$ , i.e.

(3) $$\frac{1}{N}\sum_{i=1}^{N}f(z_{i})=E[f(Z)]+\epsilon \text{, where }\epsilon ∼N(0,\omega^{2})$$

In the context of simulation-based trial design, if *Z* is the test statistic to be compared with an acceptance region Λ then the probability of acceptance under hypothesis *H* is E[I(Z∈Λ)|H] , where I(.) is the indicator function. An MC estimate of the power of a trial design under *H* can therefore be obtained given *N* test statistics $z_{1},\ldots ,z_{N}$ sampled under *H*. The steps required to simulate these statistics are described in Landau S and Stahl,^3^ and we briefly summarise them here:

1. Define the population model. This describes the underlying target population and should specify all population parameters and distributions under the hypothesis of interest.
2. Define the sampling strategy. This should specify the numbers of patients, clusters or any other sampling units in the trial and how they will be drawn from the population.
3. Define the method of analysis. For hypothesis testing, this will include defining the form of the test statistic *Z* and the acceptance region Λ.

Given each of the above elements, pseudo-random number generators can be used to simulate the recruitment, randomisation and primary outcome measure of patients under the hypothesis of interest, from which a test statistic *z_i_* can be calculated.

### 3.2. Multi-objective optimisation

A solution to the SSD problem consists of a vector of design parameters **x**. The *solution space* X is the set of all solutions. A simple SSD problem may have a one-dimensional solution space, while more complex problems may have several dimensions. Elements of **x** may include parameters defining the sample size of the trial, the acceptance region to be used in the analysis, or any other design aspect over which we have control and which may influence the trial operating characteristics. For instance, in a cluster randomised trial we could define a solution by x=(k,n) , where *k* represents the number of clusters and *n* the number of participants in each arm.

An *objective function* f(x) is a function f:X→ℝ which we wish to minimise. In a multi-objective problem with *B* objectives, we denote the vector of objective values as $y=(f_{1}(x),\ldots ,f_{B}(x))\in {\text{R}}^{B}$ . We will describe ${\text{R}}^{B}$ as the *objective space*. Extending the cluster randomised trial example, we have two objectives: minimising the number of clusters $f_{1}(x)=k$ ; and minimising the total number of participants, $f_{2}(x)=2n$ .

A *constraint function* g(x) is a function g:X→ℝ which must be less than or equal to 0 for the solution **x** to be considered feasible. For example, if type II error rate is denoted by β(x) and the nominal type II error rate is set at $\beta^{*}$ , a constraint function would be $g(x)=\beta (x)-\beta^{*}$ . We denote *C* constraint functions as $g_{j}(x),\;j=1,\ldots ,C$ . The general SSD problem can now be stated as

(4) $${\underset{x\in X}{\min}}_{\;}\;f_{i}(x),\;i=1,\ldots ,B$$

(5) $$\text{subject to}\;g_{j}(x)\leq 0,\;j=1,\ldots ,C$$

**Figure 2.**
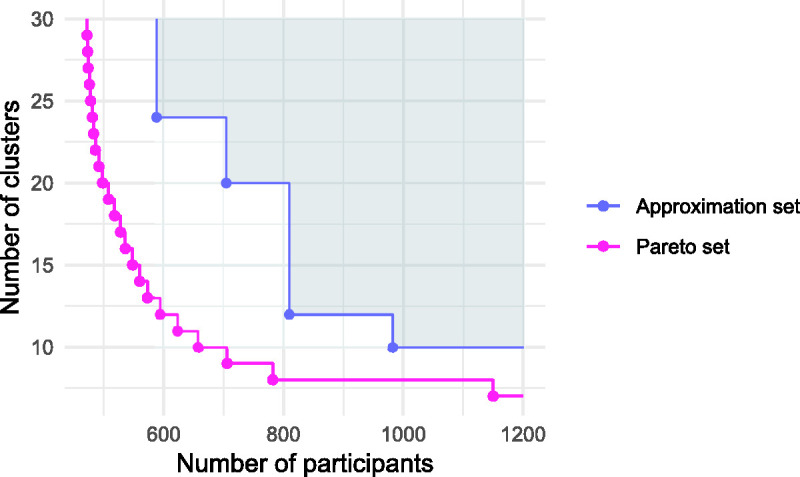
Example Pareto front $X_{p}$ and approximation set A for a cluster randomised trial design problem. The dominated hypervolume of the approximation set with respect to a reference point (cross) is the shaded area.

We denote by ≺ the relation of Pareto dominance, where a solution dominates another if it is at least as good in all respects, and better in at least one. Formally, $x_{*}≺x$ if $f_{i}(x_{*})\leq f_{i}(x)$ for i=1,…,B , and $f_{j}(x_{*})<f_{j}(x)$ for at least one *j*. For instance, $x_{a}=(n=100,k=10)≺x_{b}=(n=120,k=10)$ in our cluster randomised trial example, but $x_{a}=(n=100,k=10)⊀x_{c}=(n=80,k=13)$ . The *Pareto set* is the set of non-dominated solutions $X_{p}=\{x\in X|∄\;x_{*}\in X\;\text{s}.\text{t}.\;x_{\text{*}}≺x\}$ . An example Pareto set is plotted in Figure 2, illustrating the available trade-offs between the objectives of minimising the number of clusters and number of participants in a cluster trial.

Multi-objective optimisation seeks to find a set of solutions that are close to the true Pareto set, with every member of the set non-dominated with respect to all other members. We will refer to these as approximation sets, denoted A . That is, any set A⊂X such that $\{x\in A:\exists \;x_{*}\in A,x_{*}≺x\}=\emptyset$ is an approximation set.^29^ A set A is feasible if all constraints are satisfied by every member of A . An example feasible approximation set, plotted in Figure 2, is given by the four (2n,k) points

(6) A={(589,24), (705,20), (810,12), (982,10)}

To understand the similarity between any approximation set A and the ideal Pareto set, we measure its dominated hypervolume. This is the volume of the subspace dominated by solutions in A and bounded by a reference point *r*

(7) $$H(A)=\text{Vol}(\{y\in {\mathbb{R}}^{B}|y\text{ is dominated by some }y_{*}\in A\text{ and }y≺r\})$$

The specific choice of *r* is not important, providing it is larger than anticipated worst-case objective values. The largest possible hypervolume of any feasible approximation set A is achieved by the true Pareto set $X_{p}$ . We can therefore frame the multi-objective optimisation problem as finding the feasible approximation set A with largest hypervolume. Taking a reference point of r=(1200,30) (marked by the cross in Figure 2), our example approximation set has a dominated hypervolume of 9202. This can be compared with that of the Pareto set, at 14501. We would expect the approximation set to converge to the Pareto set as the number of optimisation iterations increases.

## 4. Simulation-based sample size determination

### 4.1. Overview

The proposed method is based on the efficient global optimisation algorithm.^35^ For clarity, we will describe the algorithm in the context of an SSD problem with a single constraint function, denoted g(x) , which must be estimated using simulation. The more general case of several constraints will follow. The initial step is to select a number of potential solutions to the SSD problem $X_{E}=(x^{\left(1\right)},\ldots ,x^{\left(E\right)})$ and evaluate the constraint function at each of these points, giving $y_{E}=(g(x^{\left(1\right)}),\ldots ,g(x^{\left(E\right)}))$ . A Gaussian process regression model is then fitted to the data, relating the solutions $X_{E}$ to the estimates $y_{E}$ and providing an approximation of the constraint function *g*. The solution $x_{*}$ which has the largest expected improvement EI(x) according to the predictions of the GP model, is then found. This solution is evaluated to obtain $y_{*}$ . This new data is then used to update the GP model, which is then used again to find the next solution to evaluate. The algorithm can be repeated until either the computational resources available have been exceeded, or until no further improvements are being obtained. Algorithm 1 is summarised below.

Algorithm 1 Efficient global optimisation^35^

1: Compute MC estimates $y_{E}=(g(x^{\left(1\right)}),\ldots ,g(x^{\left(E\right)}))$
**2: While** computation budget not exhausted **do**
3: Regress $y_{E}$ on $X_{E}=(x^{\left(1\right)},\ldots ,x^{\left(E\right)})$
4: Find $x_{*}=\arg\max EI(x)$
5: Compute MC estimate $y_{*}=g(x_{*})$ and add to $y_{E},\;X_{E}$
6: Update the computational budget
**7: End while**

 The process of computing MC estimates used in steps (1) and (5) has already been described in section 3.1. In what follows we will first consider step (3), describing Gaussian process regression models and outlining how they can be fitted and used to make predictions. The notion of expected improvement in step (4) will then be defined for the constrained multi-objective problems we are concerned with. Finally, we cover the remaining aspects of implementation.

### 4.2. Gaussian process regression

Consider a set of points $X_{E}=\{x^{\left(1\right)},\ldots ,x^{\left(E\right)}\}\subset X$ at which an expensive function *g* will be estimated using the Monte Carlo method. Consider also some other point x*∈XE where we are interested in making a prediction of $g(x_{*})$ . The value of *g* at each point in $\left\{X_{E},x_{*}\right\}$ is initially unknown, but can be modelled by a Gaussian process (GP).

In using a GP we assume that our belief regarding the values of *g* can be represented by a multivariate normal distribution. Prior to computing any estimates of *g*, we assume that the mean function of this multivariate normal is equal to zero.^a^ We write the covariance matrix of the distribution as

(8) $$\left(\begin{array}{cc} K(X_{E},X_{E}) & K(X_{E},x_{*})\\ K(x_{*},X_{E}) & K(x_{*},x_{*}) \end{array}\right)$$

where $K(X_{E},X_{E})$ is the *E *×* E* covariance matrix for the points $X_{E},\;\;k_{*}=K(X_{E},x_{*})=K(x_{*},X_{E})$ is the *E*-length vector of covariances between $X_{E}$ and $x_{*}$ and $K(x_{*},x_{*})$ is the variance at $x_{*}$ .

Given this prior distribution, we compute the MC estimates $y^{\left(1\right)},\ldots ,y^{\left(E\right)}$ at each point in $X_{E}$ . From equation (3), $y^{\left(i\right)}=g(x^{\left(i\right)})+\epsilon^{\left(i\right)}$ where $\epsilon^{\left(i\right)}$ is a zero-mean normally distributed error term with standard deviation $\omega^{\left(i\right)}$ . We denote by Δ the *E *×* E* diagonal matrix where the *i*th entry is ${\left[\omega^{\left(i\right)}\right]}^{2}$ . The distribution of $g(x_{*})$ conditional on the observed **y** can be shown to be normal with mean $k_{*}^{⊤}{\left(K+\Delta \right)}^{-1}y$ and variance $k(x_{*},x_{*})-k_{*}^{⊤}{\left(K+\Delta \right)}^{-1}k_{*}$ .^25^ Thus, given a prior covariance matrix of the form (8) and some MC estimates of *g* at the points $X_{E}$ , a conditional predictive distribution of $g(x_{*})$ can be found. It is this distribution which will be used in the optimisation algorithm when deciding which solution should next be evaluated.

The predictive distributions are influenced by the prior covariance matrix (8). The matrix is populated using a covariance function (or *kernel*), k(x,x′):X×X→ℝ . This function must be symmetric and positive definite for the covariance matrix to have the same properties. One such covariance function is the squared exponential, which has the form

(9) $$k(x,x\prime )=\sigma \exp\left(-\sum_{j=1}^{D}\frac{{\left(x_{j}-x\prime_{j}\right)}^{2}}{\lambda_{j}^{2}}\right)$$

By using covariance functions of this form we will obtain a Gaussian process which is infinitely differentiable over X and thus very smooth. This would appear to be a reasonable restriction to place upon the power functions we are interested in. In order to populate the covariance matrix we must choose values of the hyperparameters $\theta =(\sigma ,\lambda_{1},\ldots ,\lambda_{D})$ . We do this by numerically optimising the log marginal likelihood

(10) $$\log p(y|X_{E},\theta )=-\frac{1}{2}y^{⊤}{\left[K+\Delta \right]}^{-1}y-\frac{1}{2}\log|K+\Delta |-\frac{n}{2}\log2\pi$$

considered as a function of θ .^25^ Fitting a GP model by maximum likelihood in this manner can be done using the function km in the R package DiceKriging, as illustrated in the supplemental material.

**Figure 3.**
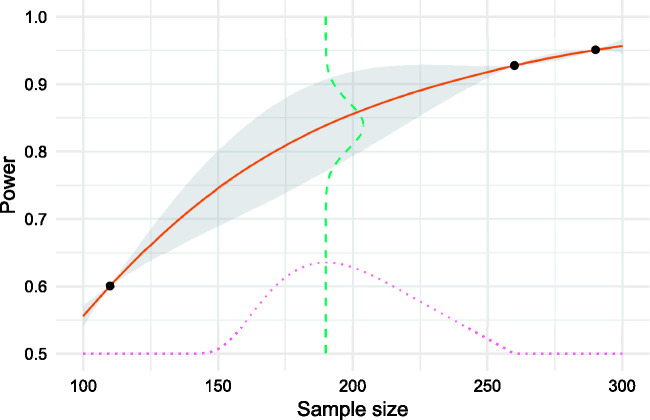
A Gaussian process model of a power function over a one-dimensional sample size (solid line) based on three evaluations. Uncertainty is shown as the shaded area. Expected improvement (dotted line) is maximised at a sample size of 190, where the predicted power is normally distributed around a mean estimate of 0.84 (dashed line).

An illustration of a Gaussian process regression model of a power function in one dimension is given in Figure 3. The power of three different choices of sample size has been calculated and a GP model fitted to the results. The figure illustrates how the uncertainty in the model predictions (shaded area) increases the further we are from a point which has been evaluated. The GP prediction of power at a sample size of *n *=* *190, shown as a dashed line, is normally distributed with mean 0.84 and standard deviation 0.035.

### 4.3. Expected improvement

At any given point during the optimisation process we can obtain an approximation set A based on the set of solutions which have been evaluated up to that point. If a new point $x_{*}$ is considered feasible, a new approximation set $A_{*}$ will be identified. The improvement resulting from the evaluation of $x_{*}$ is the difference in the dominated hypervolumes

(11) $$I=H(A_{*})-H(A)$$

Prior to evaluation, we do not know if the point $x_{*}$ will be considered feasible. We therefore modify *I* to account for the probability that $x_{*}$ will be considered feasible after the MC estimates have been obtained. This probability can be estimated using the GP regression methodology described in section 4.2. A GP model of unknown constraint function *g* will describe our current belief about the value of *g* at $x_{*}$ using a normal distribution with mean *m* and variance *s*^2^ $g(x_{*})∼N(m,s^{2})$ , and we will consider the point $x_{*}$ feasible if the upper 100×p % quantile of this distribution is below 0. We denote this quantile as

(12) $$q(x_{*})=m+\Phi^{-1}(p)s$$

where Φ is the standard normal cumulative distribution. Following an evaluation of $x_{*}$ the GP model will be updated and the quantile revised to $q_{+}(x_{*})$ . Before the evaluation the value of $q_{+}(x_{*})$ is unknown, but it is shown in Picheny and Ginsbourger^30^ that its predictive distribution is $q_{+}(x_{*})∼N(m_{+},s_{+}^{2})$ where

(13) $$m_{+}=m+\Phi^{-1}(p)\sqrt{\frac{\omega^{2}s^{2}}{\omega^{2}+s^{2}}}$$

(14) $$s_{+}^{2}=\frac{{\left[s^{2}\right]}^{2}}{\omega^{2}+s^{2}}$$

and *ω* is the MC error of the planned evaluation, estimated as m(1−m)/N where *N* is the number of MC samples to be used. The predictive distribution can then be used to calculate the probability that the point $x_{*}$ will be considered feasible following its evaluation. Following Sasena et al.,^28^ we multiply the theoretical improvement *I* by this probability, thus penalising candidate solutions with a low chance of satisfying the constraint. This then gives us our expected improvement measure *EI*, where

(15) $$\text{Expected Improvement }EI(x_{*})=[H(A_{*})-H(A)]\prod_{j=1}^{C}\Phi \left(\frac{-m_{j,+}}{s_{j,+}}\right)$$

Note that we include a penalty term for all j=1,…,C constraint functions. This maximisation problem is in itself complex, with a potentially large number of local maxima. We therefore use the particle swarm optimisation algorithm as implemented in the R package pso,^36^ designed to avoid becoming trapped in local maxima, to solve this sub-problem.

An illustration of expected improvement for a single-objective problem is given in Figure 3. When choosing which sample size to evaluate next and aiming to find the lowest per-arm sample size with at least 80% power, we balance the potential improvement over the best current solution (a sample size of 260) with the probability of constraint satisfaction. In this case, we would choose to evaluate the sample size of 190, estimated by the GP model to have a power of 84%.

### 4.4. Fixed space-filling design

An alternative to the EGO algorithm is to evaluate a number of solutions chosen using a space-filling experimental design, and use these to construct an approximation set. Specifically, we construct a Sobol sequence (a low-discrepancy sequence which can be thought of as a quasi-random uniform distribution) and estimate the type II error rate at each of these solutions using *N* MC samples. For each point a confidence interval based on the MC error can then be calculated, and any points where the interval was not entirely below the nominal value discarded. Of those that remained, any dominated solutions are discarded to leave an approximation set. We will refer to this as the ‘fixed design’ method.

### 4.5. Implementation

To apply Algorithm 1 in practice we must first choose the initial set of points to be evaluated, $X_{E}$ . One recommendation is to use a space-filling design with 10 points for each dimension of the solution space, and to allocate between 30% and 50% of the total computation budget to their evaluation.^37^ To select the location of the points in $X_{E}$ we use the space-filling Sobol sequence generated using the R package randtoolbox.^38^ The number of iterations and the number of MC samples *N* used at each iteration must also be chosen. Given a total computational budget in terms of MC samples, the choice of these values should account for the fact that fitting GP regression models in R to more than around 800 points is currently infeasible.^39^ The choice of the computational budget itself is not critical since, after terminating the algorithm, it can be simply restarted from its final state. This may be sensible if, for example, the trajectory of solution quality does not appear to have plateaued at termination.

As the algorithm depends on GP regression models, it can be helpful to assess the fit of these models. One approach is to regularly plot the predicted mean and standard deviation in one or two dimensions, centred at the last evaluated point. Poor model fit could be identified if the mean function is not, for example, strictly increasing as expected. We can also contrast the predicted function values with the obtained function values at each iteration, halting the algorithm if a large and unexpected discrepancy in these values is observed.

We have implemented the proposed framework in R, partly due to the availability of robust and efficient R packages for fitting Gaussian process models (DiceKriging^26^) and for global optimisation (pso^36^). Using R also provides flexibility in terms of the user-written simulation routines by facilitating various complicated analysis procedures, e.g. multilevel modelling through lme4.^40^ The implementation is provided in the supplementary material and online at https://github.com/DTWilson/Bayes_opt_SSD, where we show how it can be applied to solve a number of example SSD problems.

## 5. Illustration and evaluation

### 5.1. Application to a hypothetical example

We begin by showing how the proposed method can be applied to design a hypothetical two-arm cluster randomised trial with a continuous, normally distributed outcome, *k* clusters in each arm, and *m* participants in each cluster. The outcome of participant *i* in cluster *j* is modelled as

(16) $$y_{ij}=\beta_{0}+\beta_{1}t_{i}+u_{j}+e_{i}$$

(17) $$u_{j}∼N(0,\sigma_{B}^{2})$$

(18) $$e_{i}∼N(0,\sigma_{W}^{2})$$

where *t_i_* = 1 if patient *i* is in the intervention arm, and *t_i_* = 0 otherwise. The trial will be analysed using a two-sample *t*-test comparing the cluster sample means

$${\bar{y}}_{j}=\frac{1}{m}\sum_{i=1}^{m}y_{ij}$$

in the two arms. We assume the nuisance parameters are known and equal to $\sigma_{W}^{2}=0.95,\sigma_{B}^{2}=0.05$ .

To apply the proposed method, we define *k* and n=m*k as design parameters and search over the ranges k∈[10,100] and n∈[100,500] . We wish to minimise the two objectives $f_{1}(x)=2n$ (the total number of participants) and $f_{2}(x)=2k$ (the total number of clusters). Fixing the type I error rate at 0.025 (one-sided), we can then use simulation to estimate the type II error rate and find an approximation set of solutions which give a value no more than the nominal 0.1. We initialised the optimisation algorithm by generating a Sobol sequence of size 20 and computing MC estimates of power for each point using *N *=* *100 samples. Following this, 30 iterations of the algorithm were applied, with *N *=* *100 samples used at each iteration. To explore the effect of increasing the computational budget, we then continued with another 150 iterations of the algorithm. For comparison, we found an alternative approximation set using the approach of section 4.4, evaluating 50 solutions in a fixed space-filling design. Finally, we note that in this example power can be calculated analytically (by noting that the variance of the cluster means will equal $\sigma_{B}^{2}+\sigma_{W}^{2}/m$ ), and so we can find the true Pareto set and compare the three approximation sets against it.

**Figure 4.**
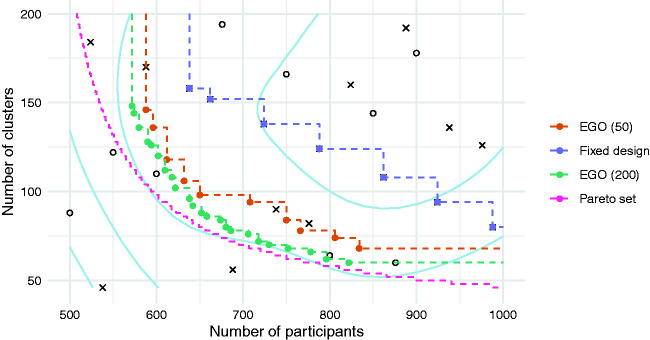
Objective values of solutions in the initial set $X_{E}$ (open circles), subsequent iterations of the algorithm (crosses), and those in the final approximations of the Pareto set (filled circles). The approximation sets are obtained using the EGO algorithm for 50 and 200 evaluations, and using a fixed design of size 50). Contours represent the mean function of the final GP model.

In Figure 4, we plot the 50 solutions evaluated over the course of the EGO algorithm, distinguishing between those in the initial design $X_{E}$ , those which were subsequently evaluated during the iterative phase of the algorithm, and those which together form the final approximation set. The contours of the mean function of the final GP model are also shown. We also plot the approximation front obtained using the comparator approach, the true Pareto set, and the approximation set obtained by running a further 150 iterations of the EGO algorithm.

We find that the EGO algorithm leads to a much better approximation set than the comparator, for the same computation budget. For example, the fixed design method suggests that in order to limit the number of clusters to 80, a total of 988 participants will be required. In contrast, the EGO method shows that a total sample size of only 766 is needed. The true minimal sample size required for 80 clusters is 649 participants, showing that while the EGO algorithm can substantially improve upon the simpler alternative, it has still suggested an approximation set that is some distance from the Pareto set. As shown in Table 1, all the suggested solutions are adequately powered with a true type II error rate *β* less than the nominal 0.1. Increasing the computational budget by applying a further 150 iterations of the algorithm moves the approximation set closer to the Pareto set, as would be expected.

**Table 1.** Approximation sets for the hypothetical example obtained using the EGO algorithm and the fixed design approach, with 50 evaluations each. Solutions are defined by the total number of participants, 2*n*, and the total number of clusters, 2*k*. The true type II error rate *β* is shown alongside with the estimate $\hat{\beta }$ obtained using *N *=* *100 MC samples.

EGO				Fixed design			
2*n*	2*k*	$\hat{\beta }$ (s.e.)	*β*	2*n*	2*k*	$\hat{\beta }$ (s.e.)	*β*
834	68	0.06 (0.026)	0.073	988	80	0.04 (0.019)	0.039
806	74	0.07 (0.025)	0.070	924	94	0.05 (0.019)	0.036
766	78	0.11 (0.026)	0.073	862	108	0.05 (0.019)	0.036
750	84	0.10 (0.026)	0.070	788	124	0.03 (0.02)	0.040
708	94	0.06 (0.026)	0.071	724	138	0.02 (0.021)	0.047
650	98	0.13 (0.028)	0.083	662	152	0.05 (0.023)	0.056
632	106	0.07 (0.028)	0.083	638	158	0.05 (0.024)	0.061
612	118	0.07 (0.028)	0.083				
596	136	0.07 (0.027)	0.080				
588	146	0.08 (0.027)	0.080				

EGO: efficient global optimisation.

### 5.2. Simulation study

To understand the variability in performance of the proposed method, we conducted a simulation study based on the preceding hypothetical example in section 5.1. We applied the method as before, with 20 initial evaluations at points determined by a Sobol sequence, followed by 30 iterations of the algorithm. At each evaluation, *N *=* *100 MC samples were used when estimating power. We repeated this process 500 times. For comparison, we also applied the fixed design approach by evaluating 50 solutions chosen by a Sobol sequence, discarding those with an estimated type II error rate greater than the nominal 0.1, and finding the approximation set from the remaining solutions. We repeated this process 500 times, again using N=100 MC samples at each evaluation. We then extended the fixed design from size 50 to size 200, 400, 600, 800 and 1000. For each approximation set obtained, we recorded the dominated hypervolume and the number of solutions it contains. We also calculated the true type II error rate for each solution, and recorded the proportion of solutions in the approximation set which had a true type II error rate no more than the nominal 0.1.

**Figure 5.**
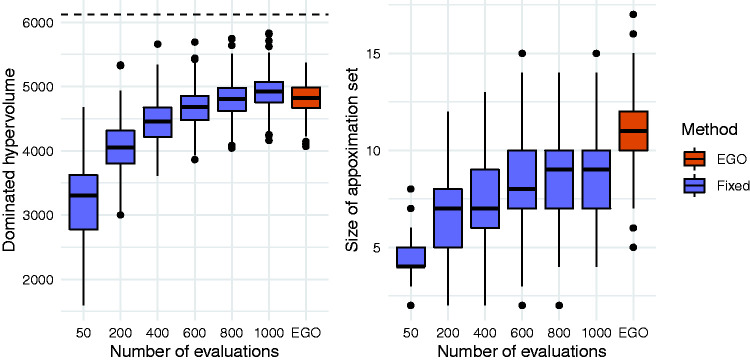
Distributions of dominated hypervolumes (left) and size (right) of approximation sets obtained using the EGO method and the fixed design method. The dashed horizontal line on the left hand plot indicates the dominated hypervolume of the true Pareto set.

We plot the distribution of the dominated hypervolumes in Figure 5. Comparing the EGO and fixed design methods when each uses 50 evaluations in total, we find that the average dominated hypervolumes are 4824 and 3243, respectively. There is some overlap in the distributions. For example, 92% of EGO runs led to a dominated hypervolume greater than 4500, compared with 1% of fixed design runs. Increasing the number of evaluations in the fixed design improves the solution quality as expected, but to obtain an average performance similar to the EGO algorithm the number of evaluations must be increased to between 800 and 1000.

Figure 5 shows that the approximation sets are typically larger than those obtained using fixed designs, with an average of 10.9 solutions. In comparison, a fixed design of size 50 had an average of 4.4. This suggests that as well as producing approximation sets of consistently higher quality, the EGO approach will also provide more choice for trading off the two conflicting objectives. The vast majority (98%) of EGO approximation sets contained only valid solutions with a true type II error rate of at most 0.1. For the fixed design method, this proportion was similar when 50 solutions were evaluated but decreased as the number of evaluations increased. For fixed designs of size 1000, 63% of approximation sets contained at least one invalid solutions, although there were at most three invalid solutions in any one approximation set.

We also investigated the effect of changing *E*, the number of solutions evaluated initially before the iterative part of the EGO algorithm. Keeping the total number of evaluations at 50, we considered *E *=* *10 and 30 in addition to the case *E *=* *20 already presented. As before, the algorithm was run 500 times and the dominated hypervolume of the final approximation set was recorded. We found little difference between setting *E *=* *10 (mean 4823) and *E *=* *20 (mean 4824). When the number of initial evaluations was increased to *E *=* *30, we found a statistically significant decrease in the mean dominated hypervolume to 4761. We can therefore conclude that, in this example, the recommendations referenced in section 4.5 (setting *E* to ten times the dimensions of the solutions space and representing 30% to 50% of the total computational budget) work well.

### 5.3. Application to the motivating example

Recall that the PACE trial measured two primary endpoints, one relating to fatigue (denoted as *F*) and one to disability (denoted as *D*). Our model of the continuous response of the *i*th participant for endpoint r∈{F,D} can be written, using the multilevel model notation of Goldstein,^41^ as

(19) $$y_{i}^{r}=\beta_{0}^{r}+\beta_{1}^{r}t_{i}+u_{\text{therapist}(i)}^{r}t_{i}+v_{\text{doctor}(i)}^{r}+e_{i}^{r}$$

Correlation between the two endpoints is modelled by simulating bivariate residuals $\left(e_{i}^{F},e_{i}^{D}\right)$ from a joint normal distribution with correlation *ρ_W_* and marginal variances $\sigma_{W}^{2}$ . We also allow for correlation between the random effects associated with each therapist and doctor. These are simulated according to the bivariate normal distributions

$$\begin{array}{c} (u_{\text{therapist}(i)}^{F},u_{\text{therapist}(i)}^{D})∼N\left({\left(0,0\right)}^{T},\;\left(\begin{array}{cc} \sigma_{T}^{2} & \rho_{T}\sigma_{T}^{2}\\ \rho_{T}\sigma_{T}^{2} & \sigma_{T}^{2} \end{array}\right)\right)\\ (v_{\text{doctor}(i)}^{F},v_{\text{doctor}(i)}^{D})∼N\left({\left(0,0\right)}^{T},\;\left(\begin{array}{cc} \sigma_{D}^{2} & \rho_{D}\sigma_{D}^{2}\\ \rho_{D}\sigma_{D}^{2} & \sigma_{D}^{2} \end{array}\right)\right) \end{array}$$

For the purposes of power calculations we must make some assumptions about the various nuisance parameter values. We set the second level variance components to $\sigma_{T}^{2}=0.19,\sigma_{D}^{2}=0.37$ and $\sigma_{W}^{2}=3.29$ in order to give a variance partition coefficient of $\sigma_{D}^{2}/(\sigma_{D}^{2}+\sigma_{W}^{2})=0.1$ in the control arm, a typical value in this setting. Similarly, the variance partition coefficient for between-therapist variance is then $\sigma_{T}^{2}/(\sigma_{T}^{2}+\sigma_{D}^{2}+\sigma_{W}^{2})=0.05$ , and for between-doctor variation, $\sigma_{D}^{2}/(\sigma_{T}^{2}+\sigma_{D}^{2}+\sigma_{W}^{2})=0.095$ . We set all correlations equal at $\rho_{W}=\rho_{T}=\rho_{D}=\rho_{D}=0.9$ , reflecting a situation where both a patient’s responses and the individual therapist and doctor effects and very similar for both the fatigue and disability outcomes. We want to simulate the power of the trial under the alternative hypotheses $H_{1}:\beta_{1}=1.10$ . Note that this corresponds to a treatment effect standardised with respect to the total standard deviation in the intervention arm of $1.10/\sqrt{\left(0.19+0.37+3.29\right)}=0.56$ .

Our design parameters (together with the ranges considered) are the total sample size in the intervention arm, denoted *n*_1_ (50 to 100); the number of therapists, *k* (2 to 10); the allocation ratio relating the total number of participants in each arm, $r=n_{0}/n_{1}$ (0.5 to 1.5); the number of doctors, *j* (3 to 20); and the nominal type I error rate to be used in the hypothesis tests, *a* (0.05 to 0.2). The three objective functions to be minimised are $f_{1}(x)=n_{1}+rn_{1},\;f_{2}(x)=k$ and $f_{3}(x)=j$ . The two constraints to be satisfied are $g_{1}(x)=\beta (x)-0.1$ and $g_{2}(x)=\alpha (x)-0.2$ .

**Figure 6.**
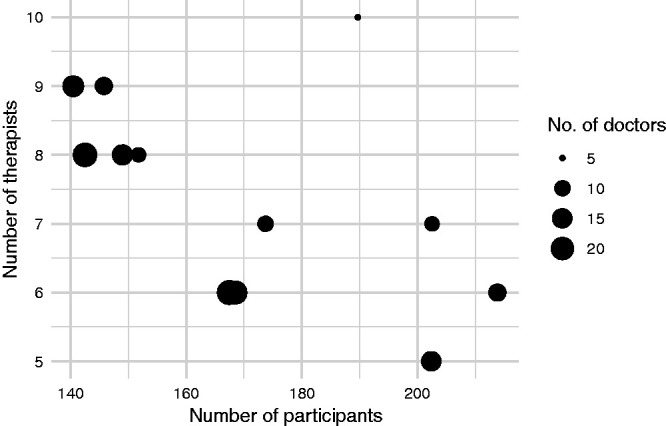
Objective values of the approximation set obtained following 50 iterations of the algorithm for the motivating example.

We initialised the algorithm by generating a Sobol sequence of size 50 and computing MC estimates of power for each point using *N *=* *100 MC samples. After 150 iterations of the algorithm, an approximation set containing 12 solutions was obtained. The algorithm took 2 h and 36 min to run on a PC with a 2.60 GHz processor and 8GB RAM. The objective values of these solutions are illustrated in Figure 6, with full details provided in Table 2. The total number of participants ranged from 140 to 214; of therapists, from 5 to 10; and of doctors, from 5 to 23. Nominal type I error rates ranged from 0.09 to 0.14, all some way below the actual constraint value of 0.2. We calculated precise MC estimates (using $N=50^{4}$ samples) of both type I and II error rates for each solution in the approximation set. As shown in Table 2, type II error rates all appear to be around or slightly below the constraint of 0.1. This demonstrates the GP’s ability to share information of MC estimates computed at several points to increase the precision at each of them. Type I error rates, in contrast, are in some cases significantly below the constraint of 0.2. This suggests there is some potential for improvement in the approximation set by applying further iterations of the algorithm. For comparison, we use the fixed design approach, evaluating 200 solutions, but found that all of these had a type I or II error rate beyond their respective constraints.

**Table 2.** Approximation set after 50 iterations for the motivating example. Solutions are defined by the number of participants in the APT arm *n*_1_, the total number of participants across both arms *n*, the number of therapists *k*, the number of doctors *j* and the nominal type I error rates *a*. Both type I and II error rates are estimated using simulation using *N* samples, and are constrained at 0.2 and 0.1, respectively.

					$N=10^{2}$		$N=50^{4}$	
*n*_1_	*n*	*k*	*j*	*a*	*β* (s.e.)	*α* (s.e.)	*β* (s.e.)	*α* (s.e.)
94	202	5	15	0.11	0.07 (0.026)	0.12 (0.033)	0.088 (0.003)	0.17 (0.004)
94	169	6	21	0.12	0.11 (0.031)	0.13 (0.034)	0.081 (0.003)	0.179 (0.004)
94	214	6	12	0.11	0.05 (0.022)	0.21 (0.041)	0.09 (0.003)	0.157 (0.004)
84	167	6	23	0.10	0.09 (0.029)	0.12 (0.033)	0.103 (0.003)	0.147 (0.004)
79	174	7	10	0.12	0.17 (0.038)	0.13 (0.034)	0.086 (0.003)	0.171 (0.004)
95	203	7	9	0.09	0.1 (0.03)	0.16 (0.037)	0.085 (0.003)	0.134 (0.003)
75	152	8	9	0.13	0.1 (0.03)	0.12 (0.033)	0.083 (0.003)	0.175 (0.004)
78	149	8	16	0.12	0.08 (0.027)	0.21 (0.041)	0.088 (0.003)	0.171 (0.004)
76	142	8	22	0.12	0.07 (0.026)	0.17 (0.038)	0.098 (0.003)	0.156 (0.004)
80	140	9	17	0.13	0.04 (0.02)	0.2 (0.04)	0.084 (0.003)	0.176 (0.004)
81	146	9	12	0.14	0.14 (0.035)	0.18 (0.039)	0.079 (0.003)	0.181 (0.004)
97	190	10	5	0.14	0.07 (0.026)	0.27 (0.045)	0.072 (0.003)	0.178 (0.004)

## 6. Discussion

Although simulation is often required for clinical trial sample size determination, related methodology has typically assumed that there is only one parameter which we are able to adjust (the sample size); that there is only one operating characteristic which must be estimated using simulation (the power of the trial); and that our goal is to minimise only one criterion (the sample size again).^3^,^5^ In this paper, we have described a flexible approach to simulation-based SSD, which can be used for more general multi-parameter problems. The method draws on established global optimisation algorithms, which use statistical ‘surrogate’ models to solve design problems where there are several parameters to be chosen, several objectives to minimise, and several constraints to satisfy. We have illustrated how such problems arise in clinical trials of complex interventions.

The general optimisation framework we have suggested recognises that in many complex trials we are interested in minimising more than one quantity subject to constraints on operating characteristics. Problems of this sort are common in multilevel trial design,^42^ but are typically approached by first reducing the multiple objectives down to a single objective. For example, in the design of a cluster randomised trial it is common to fix the number of participants per cluster and minimise the number of clusters,^43^ or vice-versa.^44,45^ Alternatively, a function that specifies the cost of sampling at the cluster and the patient level could be specified,^46^ and the overall cost could be minimised.^47^ The latter approach has been suggested for both two-level^48^ and three-level hierarchical trial designs.^49,50^ However, the *a priori* specification of such a cost function may not always be feasible, particularly when several stakeholders are involved.^51^ The Pareto optimisation framework we have described leads to a more computationally challenging optimisation problem, but produces a set of good solutions enabling the available trade-offs between objectives to be seen and selected from.

As noted in section 1, related work in simulation-based design methodology has often focussed on a specific area of application. One advantage that brings is the relative ease with which the software can be used to solve a new problem within the same area. In contrast, our approach requires that the user provides a programme which simulates the data generation and analysis of their proposed trial design. Although some have argued that this requirement may be prohibitive in practice,^18^ it allows the user to solve their specific problem rather than some related version of it. Moreover, prior to addressing the sample size issue, modelling and simulation can help inform many other aspects of trial design, such as the patient population or the choice of endpoint.^52^ One way to assist users in writing their own simulations is to share example programmes for a range of problems, providing a starting point for the development of a new programme. We have provided some examples in the supplemental material.

When submitting a proposed design for approval by a funding body it is important that the sample size calculation is transparent and replicable. This may be achieved in the context of simulation-based SSD by supplying the simulation programme as part of the application.^5^ Given this, any reviewer should be able to re-calculate the operating characteristics of the proposed design. However, a greater challenge for the reviewer is understanding the programme and ensuring it is an accurate representation of the model in question. This requirement has partly motivated our use of R. Although significantly slower than a compiled language such as C++, it has been argued that software written in R is more transparent.^52^ Validation will be further facilitated if a simulation protocol of the sort described in Burton et al.^53^ is provided alongside the code. Future work could develop an interface for alternative statistical software such as Stata or SAS, allowing a simulation programme to be written in them and connect with the R implementation of the optimisation algorithm.

We have followed the conventional approach to clinical trial design whereby constraints on operating characteristics are set and then a constrained optimisation problem is solved. In practice the constraints are not fixed in advance, but adjusted iteratively in response to the design requirements they produce. For example, an initial nominal power of 90% may require an infeasibly large sample size, leading to a revision down to 80%. Such an iterative procedure will increase an already substantial computational burden for simulation-based design. However, note that a change to a constraint does not mean starting the process again, since any previous MC estimates can still be used when fitting the GP model(s). The sequential nature of the optimisation algorithm suggests that an interactive routine could be developed, where the user pauses the algorithm in response to the sample size requirements which are being observed, adjusts the constraints, and then continues with the optimisation.

We have defined power in relation to a specific value of the parameter of interest, the so-called MCID, and specific point estimates of any nuisance parameters. Implicit in this formulation is an assumed monotonicity of the power function with respect to the parameter of interest. That is, we assume that if the power at this point is above the nominal constraint, then power at all larger values will also be above this level. When this assumption cannot be made, the method could be applied by setting further power constraints at other points in the parameter space. This approach could also be taken with respect to any nuisance parameters, helping to guard against any error in their estimation. This will, however, increase the computational burden by requiring more simulations at each iteration of the algorithm.

The examples have demonstrated that the time required to solve a sample size determination problem can be significant, of the order of hours. Given that the majority of computational effort is expended generating MC samples when evaluating solutions, it is important that these simulation programmes are as efficient as possible. We recommend making use of code profilers such as R’s ‘Rprof’ to identify the parts of the programme that are consuming the most resources. Further efficiencies could potentially be gained by using more sophisticated methods for surrogate modelling and efficient optimisation. For example, prior knowledge such as the power function being bounded or monotonic could be incorporated into the surrogate modelling process.^29^

Numerous extensions to the proposed approach can be considered. One argument for simulation-based design is the ease with which sensitivity to model assumptions, such as the value of nuisance parameters, can be assessed.^3^ Future work could consider how a systematic assessment of sensitivity to nuisance parameters could be conducted, given a proposed trial design. Such investigations fall under the heading of *uncertainty quantification* and can be carried out using GP regression and associated techniques.^54^ A further extension could consider Bayesian approaches to trial design, including hybrid Bayesian-frequentist assurances,^55^ fully Bayesian measures such as average coverage criterion^56^ and decision-theoretic methods.^57^ Aside from very simple cases involving only conjugate analyses, evaluating these Bayesian criteria will generally require simulation^55^ and so optimal design may benefit from the efficient methods discussed here. Complex SSD problems are also common in the area of adaptive designs, which can aim to minimise the expected sample size under several different hypotheses and over a number of stopping rule parameters.^19^ Extending the proposed methods to such problems would require using surrogate models to approximate the objective functions, as opposed to only the constraints.

In conclusion, efficient optimisation algorithms based on surrogate models of expensive operating characteristic functions can be used to solve complex clinical trial sample size determination problems. By using these methods we can avoid making unrealistic simplifying assumptions at the trial design stage, both in terms of the statistical model underlying the trial and of the nature of the design problem.

## Supplemental Material

sj-zip-1-smm-10.1177_0962280220975790 - Supplemental material for Efficient and flexible simulation-based sample size determination for clinical trials with multiple design parameters Supplemental material, sj-zip-1-smm-10.1177_0962280220975790 for Efficient and flexible simulation-based sample size determination for clinical trials with multiple design parameters by Duncan T Wilson, Richard Hooper, Julia Brown, Amanda J Farrin and Rebecca EA Walwyn in Statistical Methods in Medical Research

## Note

a This is not a restrictive assumption. After observing estimates of the function *g* and updating the GP model to account for these, the mean function can take on non-zero values.

## References

[1] Cook JA Julious SA Sones W , et al. DELTA^2^ guidance on choosing the target difference and undertaking and reporting the sample size calculation for a randomised controlled trial. Trials 2018; 19: 606. 10.1186/s13063-018-2884-0 PMC6218987 30400926

[2] Arnold BF Hogan DR Colford JM , et al. Simulation methods to estimate design power: an overview for applied research. BMC Med Res Methodol 2011; 11: 94. 10.1186/1471-2288-11-94 PMC3146952 21689447

[3] Landau S Stahl D. Sample size and power calculations for medical studies by simulation when closed form expressions are not available. Stat Methods Med Res 2013; 22: 324–345. 22491174 10.1177/0962280212439578

[4] Feng Z Grizzle JE. Correlated binomial variates: properties of estimator of intraclass correlation and its effect on sample size calculation. Stat Med 1992; 11: 1607–1614. 1439364 10.1002/sim.4780111208

[5] Hooper R. Versatile sample-size calculation using simulation. STATA J 2013; 13: 21–38.

[6] Schoenfeld DA Borenstein M. Calculating the power or sample size for the logistic and proportional hazards models. J Stat Comput Simul 2005; 75: 771–785.

[7] Grieve AP Sarker SJ. Simulation-based sample-sizing and power calculations in logistic regression with partial prior information. Pharmaceut Stat 2016; 15: 507–516. 10.1002/pst.1773 27588379

[8] Sutton AJ Cooper NJ Jones DR , et al. Evidence-based sample size calculations based upon updated meta-analysis. Stat Med 2007; 26: 2479–2500. 16981184 10.1002/sim.2704

[9] Fedorov V Jones B. The design of multicentre trials. Stat Methods Med Res 2005; 14: 205–248. 15969302 10.1191/0962280205sm399oa

[10] Baio G Copas A Ambler G , et al. Sample size calculation for a stepped wedge trial. Trials 2015; 16: 354. 10.1186/s13063-015-0840-9 PMC4538764 26282553

[11] Hooper R Teerenstra S de Hoop E , et al. Sample size calculation for stepped wedge and other longitudinal cluster randomised trials. Stat Med 2016; 35: 4718–4728. 27350420 10.1002/sim.7028

[12] Reich NG Myers JA Obeng D , et al. Empirical power and sample size calculations for cluster-randomized and cluster-randomized crossover studies. PLOS One 2012; 7: 1–7. 10.1371/journal.pone.0035564 PMC3338707 22558168

[13] Wilson DT Walwyn RE Brown J , et al. Statistical challenges in assessing potential efficacy of complex interventions in pilot or feasibility studies. Stat Methods Med Res 2015; 25: 997–1009. 26071430 10.1177/0962280215589507

[14] Feiveson AH. Power by simulation. Stata J 2002; 2: 107–124.

[15] Browne WJ Lahi MG Parker RM. A guide to sample size calculations for random effect models via simulation and the MLPowSim software package, http://citeseerx.ist.psu.edu/viewdoc/download?doi=10.1.1.408.6314\&rep=rep1\&type=pdf (2009, accessed 25 November 2020).

[16] Williams MS Ebel ED Wagner BA. Monte Carlo approaches for determining power and sample size in low-prevalence applications. Prev Vet Med 2007; 82: 151–158. 17590459 10.1016/j.prevetmed.2007.05.015

[17] Jung SH. Sample size calculation for paired survival data: a simulation method. J Stat Comput Simul 2008; 78: 85–92.

[18] Kontopantelis E Springate DA Parisi R , et al. Simulation-based power calculations for mixed effects modeling: ipdpower in stata. J Stat Softw 2016; 74.

[19] Wason JMS Jaki T. Optimal design of multi-arm multi-stage trials. Stat Med 2012; 31: 4269–4279. 22826199 10.1002/sim.5513

[20] Deb K Pratap A Agarwal S , et al. A fast and elitist multiobjective genetic algorithm: NSGA-II. IEEE Trans Evol Comput 2002; 6: 182–197.

[21] Mersmann O. mco: multiple criteria optimization algorithms and related functions. R package version 1.0-15.1, https://CRAN.R-project.org/package=mco (2014, accessed 25 November 2020).

[22] Sacks J Welch WJ Mitchell TJ , et al. Design and analysis of computer experiments. Stat Sci 1989; 4: 409–423.

[23] Santner TJ Williams BJ Notz WI. The design and analysis of computer experiments. New York: Springer, 2003.

[24] Krige D. A statistical approach to some basic mine valuation problems on the Witwatersrand. J South Afr Inst Mining Metall 1951; 52: 119–139.

[25] Rasmussen CE Williams CKI. Gaussian processes for machine learning. Boston, MA: MIT Press, 2006.

[26] Roustant O Ginsbourger D Deville Y. DiceKriging, DiceOptim: two R packages for the analysis of computer experiments by Kriging-based metamodeling and optimization. J Stat Softw 2012; 51: 1–55.23504300

[27] Jones DR. A taxonomy of global optimization methods based on response surfaces. J Global Optim 2001; 21: 345–383.

[28] Sasena MJ Papalambros P Goovaerts P. Exploration of metamodeling sampling criteria for constrained global optimization. Eng Optim 2002; 34: 263–278.

[29] Emmerich MT Deutz AH Klinkenberg JW. Hypervolume-based expected improvement: Monotonicity properties and exact computation. In: *2011 IEEE congress on evolutionary computation (CEC)*, 2011, pp. 2147–2154. New York: IEEE.

[30] Picheny V Ginsbourger D. Noisy Kriging-based optimization methods: a unified implementation within the DiceOptim package. Comput Stat Data Anal 2014; 71: 1035–1053.

[31] White P Sharpe M Chalder T , et al. Protocol for the pace trial: a randomised controlled trial of adaptive pacing, cognitive behaviour therapy, and graded exercise as supplements to standardised specialist medical care versus standardised specialist medical care alone for patients with the chronic fatigue syndrome/myalgic encephalomyelitis or encephalopathy. BMC Neurol 2007; 7: 6. 17397525 10.1186/1471-2377-7-6 PMC2147058

[32] White P Goldsmith K Johnson A , et al. Comparison of adaptive pacing therapy, cognitive behaviour therapy, graded exercise therapy, and specialist medical care for chronic fatigue syndrome (pace): a randomised trial. Lancet 2011; 377: 823–836. 21334061 10.1016/S0140-6736(11)60096-2 PMC3065633

[33] Walwyn REA Roberts C. Therapist variation within randomised trials of psychotherapy: implications for precision, internal and external validity. Stat Methods Med Res 2010; 19: 291–315. 19608603 10.1177/0962280209105017

[34] Senn S Bretz F. Power and sample size when multiple endpoints are considered. Pharmaceut Stat 2007; 6: 161–170. 10.1002/pst.301 17674404

[35] Jones DR Schonlau M Welch WJ. Efficient global optimization of expensive black-box functions. J Global Optim 1998; 13: 455–492.

[36] Bendtsen C. pso: particle swarm optimization. R package version 1.0.3, https://CRAN.R-project.org/package=pso (2012, accessed 25 November 2020)

[37] Picheny V Ginsbourger D Richet Y. Noisy expected improvement and on-line computation time allocation for the optimization of simulators with tunable fidelity. In: *2nd international conference on engineering optimization*, Lisbon, Portugal, 6-9 September 2010.

[38] Dutang C Savicky P. randtoolbox: generating and testing random numbers. R package version 1.17, 2015.

[39] Chevalier C Picheny V Ginsbourger D. KrigInv: an efficient and user-friendly implementation of batch-sequential inversion strategies based on Kriging. Comput Stat Data Anal 2014; 71: 1021–1034.

[40] Bates D Mächler M Bolker B , et al. Fitting linear mixed-effects models using lme4. J Stat Softw 2015; 67: 1–48.

[41] Goldstein H. Multilevel statistical models. 3rd ed. London: Arnold, 2003.

[42] Hemming K Eldridge S Forbes G , et al. How to design efficient cluster randomised trials. BMJ 2017; 358. 10.1136/bmj.j3064 PMC5508848 28710062

[43] Donner A Klar N. Design and analysis of cluster randomization trials in health research. London: Arnold, 2000.

[44] Hemming K Girling A Sitch A , et al. Sample size calculations for cluster randomised controlled trials with a fixed number of clusters. BMC Med Res Methodol 2011; 11: 102. 21718530 10.1186/1471-2288-11-102 PMC3149598

[45] Eldridge SM Costelloe CE Kahan BC , et al. How big should the pilot study for my cluster randomised trial be? Stat Methods Med Res 2015; 25: 1039–1056. 26071431 10.1177/0962280215588242

[46] Hox J. Multilevel analysis: techniques and applications. Mahwah, NJ: Lawrence Erlbaum Associates, Inc., 2002.

[47] Snijders TAB Bosker RJ. Standard errors and sample sizes for two-level research. J Educ Behav Stat 1993; 18: 237–259.

[48] Raudenbush SW Liu X. Statistical power and optimal design for multisite randomized trials. Psychol Methods 2000; 5: 199–213. 10937329 10.1037/1082-989x.5.2.199

[49] van Breukelen GJ Candel MJ. Calculating sample sizes for cluster randomized trials: we can keep it simple and efficient! J Clin Epidemiol 2012; 65: 1212–1218. 23017638 10.1016/j.jclinepi.2012.06.002

[50] Teerenstra S Moerbeek M van Achterberg T , et al. Sample size calculations for 3-level cluster randomized trials. Clin Trials 2008; 5: 486–495. 18827041 10.1177/1740774508096476

[51] Joseph L Wolfson DB. Interval-based versus decision theoretic criteria for the choice of sample size. J R Stat Soc Ser D 1997; 46: 145–149.

[52] Smith MK Marshall A. Importance of protocols for simulation studies in clinical drug development. Stat Methods Med Res 2010; 20: 613–622. 20688782 10.1177/0962280210378949

[53] Burton A Altman DG Royston P , et al. The design of simulation studies in medical statistics. Stat Med 2006; 25: 4279–4292. 16947139 10.1002/sim.2673

[54] Kennedy MC O’Hagan A. Bayesian calibration of computer models. J R Stat Soc B 2001; 63: 425–464.

[55] O’Hagan A Stevens JW Campbell MJ. Assurance in clinical trial design. Pharm Stat 2005; 4: 187–201.

[56] Cao J Lee JJ Alber S. Comparison of Bayesian sample size criteria: ACC, ALC, and WOC. J Stat Plan Inference 2009; 139: 4111–4122. 25554718 10.1016/j.jspi.2009.05.041 PMC4279958

[57] Oakley JE Brennan A Tappenden P , et al. Simulation sample sizes for Monte Carlo partial EVPI calculations. J Health Econ 2010; 29: 468–477. 20378190 10.1016/j.jhealeco.2010.03.006

