# Supplementary material for: Efficient and flexible simulation-based sample size determination for clinical trials with multiple design parameters
Source: Stat Methods Med Res. 2020 Dec 2;30(3):799–815. doi: 10.1177/0962280220975790 (PMC8008419; doi:10.1177/0962280220975790)
Supplement: sj-zip-1-smm-10.1177_0962280220975790 - Supplemental material for Efficient and flexible simulation-based sample size determination for clinical trials with multiple design parameters [file sj-zip-1-smm-10.1177_0962280220975790.zip › DTW_SMMR_Bayes_opt_SSD_SM.html]

Efficient and flexible simulation-based sample size determination for clinical trials with multiple design parameters


# Efficient and flexible simulation-based sample size determination for clinical trials with multiple design parameters

#### D. T. Wilson

#### 10 September, 2020

## Introduction

This RMarkdown document contains the R code which generates the results and figures included in the manuscript of the same name, plus supplementary material. We introduce the common functions `best()` and `exp_imrprove()` in the context of the first example, and call these in the remaining two examples. Simulated data have been saved to file.

## Example 1

### Simulation

The simulation model is:

```
sim_trial <- function(x, h)
{
  ## x = design parameters, (n, k)
  ## h = hypothesis parameters, (r_t, r_d, v_w, p0, p1)
  
  n <- x[1]; k <- x[2]
  j <- 2*k
  
  r_t <- h[1]; r_d <- h[2]; v_w <- h[3]
  p0 <- h[4]; p1 <- h[5]
  
  ## Linear predicter logit terms
  lp0 <- log(p0/(1-p0)); lp1 <- log(p1/(1-p1)) - lp0
  
  ## Doctor effects
  v_d <- r_d*v_w/(1-r_d)
  doc <- cbind(seq(1,j), rnorm(j, 0, sqrt(v_d)))
  
  ## Therapist effects
  v_t <- (r_t*v_d + r_t*v_w)/(1-r_t)
  ther <- cbind(seq(0,k), c(0, rnorm(k, 0, sqrt(v_t))))

  ## Treatment group
  trt <- c(rep(0, n), rep(1, n))
  ## Patient ID
  p_id <- seq(1, 2*n)
  ## Doctor allocation and effect
  z <- rgamma(j, 1)
  ms <- round(z*2*n/sum(z))
  ms[which.max(ms)] <- ms[which.max(ms)] + 2*n - sum(ms)
  d_id <- sample(rep(doc[,1], ms))
  d_eff <- doc[d_id,2]
  ## Therapist allocation and effect
  z <- rgamma(k, 1)
  ms <- round(z*n/sum(z))
  ms[which.max(ms)] <- ms[which.max(ms)] + n - sum(ms)
  t_id <- c(rep(0, n), sample(rep(ther[2:(k+1),1], ms)))
  t_eff <- ther[t_id+1,2]
  ## Residual
  resid <- rlogis(2*n, 0, 1) ## Note - scale parameter = 1 ===> variance = 3.29
  
  data <- cbind(trt,p_id,d_id,d_eff,t_id,t_eff,resid)
  ## Latent variable
  data <- cbind(data, apply(data, 1, function(y) lp0 + y[1]*lp1 + y[4] + y[6] + y[7]))
  ## Outcome
  data <- cbind(data, data[,8] > 0)
    
  ## Analyse the data
  df <- as.data.frame(data) 
  names(df)[9] <- "y"
  
  result1 <- tryCatch({
    fit1 <- suppressMessages(glmer(y ~ trt + (0 + trt|t_id) + (1|d_id), family = "binomial", data=df))
    fit2 <- suppressMessages(glmer(y ~ (0 + trt|t_id) + (1|d_id), family = "binomial", data=df))
    p <- anova(fit1, fit2)[2,8]
  }, warning = function(war) {
    ## warning handler picks up where error was generated
    return(TRUE)
  }, error = function(err) {
    ## error handler picks up where error was generated
    return(TRUE)
  }, finally = {

  })
  
  if(result1==TRUE){
    return(c(1,1))
  } else {
    return(c(result1, 0))
  }

}

## For example,
sim_trial(x=c(n=150, k=10), h=c(r_t=0.05, r_d=0.1, v_w=3.29, p0=0.1, p1=0.25))
```

```
## [1] 0.0155764 0.0000000
```

Given this simulation, we can then evaluate the probability of failing to reject the null hypothesis of any design `x` under any hypothesis `h`, based on `N` MC samples:

```
calc_rates <- function(x, h, N)
{
  sims <- replicate(N, sim_trial(x, h) > 0.05)
  z <- sims[1,]
  ## Print out model failure rate
  print(sum(sims[2,])/N)
  ## Return proportion not rejecting null and approximate variance in estimate
  c(mean(z), var(z)/N)
}

## For example,
x <- c(n=135, k=10)
h <- c(r_t=0.05, r_d=0.1, v_w=3.29, p0=0.1, p1=0.25)

ptm <- proc.time()
calc_rates(x, h, 10)
```

```
## [1] 0.2
```

```
## [1] 0.20000000 0.01777778
```

```
proc.time() - ptm
```

```
##    user  system elapsed 
##    3.00    0.02    3.04
```

### Initial design and GP

The first step of our method is to evaluate at an initial set of design points.

```
design_space <- data.frame(name=c("n","k"), 
                           low=c(100,3), 
                           up=c(500,30)
)

DoE_num <- 20
dim <- nrow(design_space)
  
## Choose initial points using a Sobol sequence
DoE <- data.frame(sobol(DoE_num, dim))
names(DoE) <- design_space$name
for(i in 1:dim){
  DoE[,i] <-  DoE[,i]*(design_space$up[i]-design_space$low[i]) + design_space$low[i]
}
DoE[,1:2] <- round(DoE[,1:2])

## Evaluate at initial points [NOT RUN]
#N <- 100
#DoE <- cbind(DoE, t(apply(DoE, 1, calc_rates, N=N, h=h)))
#names(DoE)[3:4] <- c("beta", "beta_var")
#DoE$N <- N

## Save initial design
#saveRDS(DoE, "DoE_ex1_20_N100.Rda")
```

Given these estimates, we can model the power function over the full design space using a GP. We can then plot both the mean function and the standard deviation for each point.

```
## Load the DoE
DoE <- readRDS("./data/DoE_ex1_20_N100.Rda")

constraints <- data.frame(name=c("beta"), 
                          hyp=c("H1"), 
                          nom=c(0.1),
                          delta=c(0.975))

models <- list()
for(i in 1:nrow(constraints)){
  name <- constraints$name[i]
  models <- append(models, km(~1, design=DoE[1:dim], response=DoE[,as.character(name)], 
                             noise.var=DoE[,paste0(as.character(name), "_var")]))
  names(models)[length(models)] <- as.character(name)
}
```

```
## 
## optimisation start
## ------------------
## * estimation method   : MLE 
## * optimisation method : BFGS 
## * analytical gradient : used
## * trend model : ~1
## * covariance model : 
##   - type :  matern5_2 
##   - noise variances :
##  [1] 0.0009090909 0.0005696970 0.0021606061 0.0009888889 0.0006575758
##  [6] 0.0010666667 0.0020363636 0.0021212121 0.0008272727 0.0010666667
## [11] 0.0014909091 0.0009888889 0.0006575758 0.0002939394 0.0019909091
## [16] 0.0022979798 0.0020363636 0.0004797980 0.0019434343 0.0013575758
##   - parameters lower bounds :  1e-10 1e-10 
##   - parameters upper bounds :  700 48 
##   - variance bounds :  0.0008264125 0.1440974 
##   - best initial criterion value(s) :  24.75613 
## 
## N = 3, M = 5 machine precision = 2.22045e-16
## At X0, 0 variables are exactly at the bounds
## At iterate     0  f=      -24.756  |proj g|=      0.14084
## At iterate     1  f =      -24.931  |proj g|=      0.080307
## At iterate     2  f =      -24.931  |proj g|=      0.080767
## At iterate     3  f =      -24.931  |proj g|=      0.081015
## At iterate     4  f =      -24.931  |proj g|=      0.081055
## At iterate     5  f =      -24.931  |proj g|=       0.13606
## At iterate     6  f =      -24.931  |proj g|=       0.13607
## At iterate     7  f =      -24.931  |proj g|=       0.13609
## At iterate     8  f =      -24.931  |proj g|=       0.13612
## At iterate     9  f =      -24.931  |proj g|=       0.13618
## At iterate    10  f =      -24.933  |proj g|=       0.13626
## At iterate    11  f =      -24.935  |proj g|=        0.1364
## At iterate    12  f =      -24.943  |proj g|=       0.13661
## At iterate    13  f =      -24.959  |proj g|=       0.13686
## At iterate    14  f =      -24.983  |proj g|=       0.13691
## At iterate    15  f =      -24.994  |proj g|=      0.027588
## At iterate    16  f =      -24.994  |proj g|=       0.02756
## At iterate    17  f =      -24.994  |proj g|=      0.027557
## At iterate    18  f =      -24.994  |proj g|=      0.027557
## 
## iterations 18
## function evaluations 23
## segments explored during Cauchy searches 19
## BFGS updates skipped 0
## active bounds at final generalized Cauchy point 0
## norm of the final projected gradient 0.0275565
## final function value -24.994
## 
## F = -24.994
## final  value -24.994038 
## converged
```

```
mod <- models[[1]]

df <- expand.grid(n = seq(100,500,1), k=3:30)
pred <- predict(mod, newdata=df, type="SK")
df$beta <- pred$mean
df$sd <- pred$sd
```

```
## Plot the mean function
ggplot(df, aes(n, k, z=1-beta)) + geom_contour(aes(colour=..level..)) + geom_point(data=DoE, aes(colour=1-beta)) +
  scale_colour_gradientn(colours=rainbow(3)) + theme_minimal() + guides(colour=guide_legend(title="Power"))
```

```
## Plot the standard deviation
ggplot(df, aes(n, k)) + geom_contour(aes(z=sd, colour=..level..)) + geom_point(data=DoE) +
  theme_minimal() + guides(colour=guide_legend(title="SD"))
```

Note that the standard deviation of the GP prediction increases as we move away from evaluated points.

### Pareto fronts

Of the points which we have evaluated, We can identify which lead to sufficient power and which can be discarded because they are dominated by another feasible solution.

```
## Set up our objectives
dim <- nrow(design_space)
nobj <- 2

obj_names <- NULL
for(i in 1:nobj){
  obj_names <- c(obj_names, paste0("f",i))
}
  
objective <- function(x)
{
  c(x[1]*2/5, x[2])
}

best <- function(design_space, models, DoE, b=NULL)
{ 
  ## Return the set of current Patero optimal solutions,
  ## penalising constrain violations considering only solutions
  ## where some evaluation has actually happened
  sols <- DoE
  
  ## Get objective values
  if(nobj > 1){
    sols <- cbind(sols, t(apply(sols, 1, objective)))
  } else {
    sols <- cbind(sols, apply(sols, 1, objective))
  }
  names(sols)[(ncol(sols)-nobj+1):ncol(sols)] <- obj_names
  
  ## Penalise constraint violations
  sols$exp_pen <- 1
  for(i in 1:nrow(constraints)){
    mod <- models[[i]]
    nom <- constraints$nom[i]
    p <- predict.km(mod, newdata=sols[,1:dim, drop=F], type="SK")
    pen <- pnorm(nom, p$mean, p$sd)
    pen <- ifelse(pen < constraints[i,4], 0.0000001, 1)
    sols$exp_pen <- sols$exp_pen*pen
  }
  sols[,obj_names] <- sols[,obj_names]/sols$exp_pen
  
  ## Drop any dominated solutions
  is_nondom <- function(x, b)
  {
    i <- 1
    while(i <= nobj & nrow(b)!= 0 ){
      ## subset b to those non-dominated solutions which are less than or equal to 
      ## x in an objective
      b <- b[b[, obj_names[i] ] <= x[[ obj_names[i] ]],]
      i <- i + 1
    }
    ## If b is now empty, then des is non-dominated
    if(nrow(b)==1 | all(apply(b[,obj_names, drop=F], 2, function(x) length(unique(x)) == 1) == TRUE) ) {
      nondom <- TRUE
    } else {
      nondom <- FALSE
    }
    return(nondom)
  }
  
  nondom_sols <- sols[apply(sols, 1, is_nondom, b=sols), ]
  ## check for duplicates
  sub <- unique(nondom_sols[,obj_names, drop=F])
  
  return(nondom_sols[row.names(sub),])
}

b <- best(design_space, models, DoE)

## Add extreme points for plotting the Pareto front
b2 <- b[,1:2]
b2 <- rbind(c(min(b2[,1]), 30), b2, c(500, min(b2[,2])))

ggplot(df, aes(n, k)) + geom_contour(aes(z=beta)) + geom_point(data=DoE) +
  geom_point(data=b2[2:(nrow(b2)-1),], colour="red") + 
  geom_step(data=b2, colour="red", linetype=2) +
  theme_minimal()
```

### Expected improvement

We have identified a set of designs which we are ceratin will give nominal power and which are non-dominated. The task now is to improve this approximation set iteratively, evaluating one design at a time, updating our GP model, and then updating the set. When we choose the next point to evaluate we do so to maximimse the expected improvement which will result.

```
exp_improve <- function(design, N, p_set, models, design_space, constraints)
{
  names(design) <- design_space$name
  design <- as.data.frame(t(design))
  
  ## Get expected penalisation if we were to evaluate at design,
  ## using the models of constraint functions
  design$exp_pen <- 1
  for(i in 1:nrow(constraints)){
    mod <- models[[i]]
    nom <- constraints$nom[i]
    p <- predict.km(mod, newdata=design[,1:dim, drop=F], type="SK")
    means <- ifelse(p$mean < 0, 0, ifelse(p$mean > 1, 1, p$mean))
    mc_vars <- means*(1-means)/N
    pred_q_mean <- p$mean + qnorm(constraints[i,4])*sqrt(mc_vars*(p$sd^2)/(mc_vars+(p$sd^2)))
    pred_q_var <- ((p$sd^2)^2)/(mc_vars+(p$sd^2)) 
    design$exp_pen <- design$exp_pen*pnorm(nom, pred_q_mean, sqrt(pred_q_var))
  }
  
  ## Get objective value of design
  design <- cbind(design, t(objective(as.numeric(design))))
  names(design)[(ncol(design)-nobj+1):ncol(design)] <- obj_names
  
  ## Improvement is quantified by the number of additional
  ## solutions which would be dominated if this design was included
  ref <- objective(design_space$up)
  p_set2 <- as.matrix(p_set[,obj_names])
  current <- dominatedHypervolume(p_set2, ref)
  pos <- apply(design, 1, function(d) dominatedHypervolume(as.matrix(rbind(p_set2, d[obj_names])), ref) )
  imp <- (current-pos)*design$exp_pen
  
  ## Minimising, so keeping negative
  return(imp)
}
```

```
## Find the next point to evaluate, using a particlae swarm algorithm:
opt <- psoptim(rep(NA, 2), exp_improve, lower=design_space$low, upper=design_space$up,
               N=100, p_set=b, models=models, design_space=design_space, constraints=constraints,
               control=list(vectorize = T, max.restart=1, reltol=0.0001, trace=1))
sol <- opt$par
sol[1:2] <- round(sol[1:2])

## Evaluate the suggested point
y <- calc_rates(sol, h, N=100)
```

```
## Hard code the results
sol <- c(304, 12)
y <- c(0.06, 0.000569697)
```

The recommended evaluation is at \(n=\) 304, \(k=\) 12. Evaluating the point using \(N=100\) MC samples gives an estimated type II error rate of 0.06 (sd 0.0238683), so it appears to be feasible. We can add the new information to our records, update the GP model, and then use its predictions to confirm whether or not the new point can join the approximation set.

```
DoE <- rbind(DoE, c(sol, y, 100))

for(i in 1:length(models)){
    models[[i]] <- update(models[[i]], 
                          newX=DoE[nrow(DoE),1:2], 
                          newy=DoE[nrow(DoE), 2*i+1], 
                          newnoise.var=DoE[nrow(DoE), 2*i+2])
}
```

```
## 
## optimisation start
## ------------------
## * estimation method   : MLE 
## * optimisation method : BFGS 
## * analytical gradient : used
## * trend model : ~1
## * covariance model : 
##   - type :  matern5_2 
##   - noise variances :
##  [1] 0.0009090909 0.0005696970 0.0021606061 0.0009888889 0.0006575758
##  [6] 0.0010666667 0.0020363636 0.0021212121 0.0008272727 0.0010666667
## [11] 0.0014909091 0.0009888889 0.0006575758 0.0002939394 0.0019909091
## [16] 0.0022979798 0.0020363636 0.0004797980 0.0019434343 0.0013575758
## [21] 0.0005696970
##   - parameters lower bounds :  1e-10 1e-10 
##   - parameters upper bounds :  700 48 
##   - variance bounds :  0.0008271068 0.1432987 
##   - best initial criterion value(s) :  25.41046 
## 
## N = 3, M = 5 machine precision = 2.22045e-16
## At X0, 0 variables are exactly at the bounds
## At iterate     0  f=       -25.41  |proj g|=      0.12824
## At iterate     1  f =      -25.643  |proj g|=       0.12331
## At iterate     2  f =      -25.649  |proj g|=       0.12014
## At iterate     3  f =      -25.651  |proj g|=       0.12157
## At iterate     4  f =      -25.651  |proj g|=       0.11727
## At iterate     5  f =      -25.651  |proj g|=       0.12152
## At iterate     6  f =      -25.652  |proj g|=       0.12164
## At iterate     7  f =      -25.653  |proj g|=       0.12188
## At iterate     8  f =      -25.655  |proj g|=       0.12223
## At iterate     9  f =      -25.661  |proj g|=       0.12274
## At iterate    10  f =      -25.675  |proj g|=       0.12339
## At iterate    11  f =      -25.708  |proj g|=       0.12389
## At iterate    12  f =      -25.765  |proj g|=       0.12337
## At iterate    13  f =      -25.798  |proj g|=      0.037907
## At iterate    14  f =      -25.819  |proj g|=       0.11963
## At iterate    15  f =       -25.82  |proj g|=        0.1193
## At iterate    16  f =       -25.82  |proj g|=       0.11919
## At iterate    17  f =       -25.82  |proj g|=      0.012262
## At iterate    18  f =       -25.82  |proj g|=      0.012261
## At iterate    19  f =       -25.82  |proj g|=      0.021433
## At iterate    20  f =       -25.82  |proj g|=      0.023309
## At iterate    21  f =       -25.82  |proj g|=      0.023315
## At iterate    22  f =       -25.82  |proj g|=      0.023325
## At iterate    23  f =       -25.82  |proj g|=      0.023336
## At iterate    24  f =       -25.82  |proj g|=      0.023341
## At iterate    25  f =       -25.82  |proj g|=      0.023334
## At iterate    26  f =       -25.82  |proj g|=      0.023337
## At iterate    27  f =       -25.82  |proj g|=      0.023206
## At iterate    28  f =      -25.821  |proj g|=      0.023398
## At iterate    29  f =      -25.821  |proj g|=      0.023271
## At iterate    30  f =      -25.827  |proj g|=       0.11995
## At iterate    31  f =      -25.837  |proj g|=       0.12077
## At iterate    32  f =      -25.869  |proj g|=       0.12241
## At iterate    33  f =      -25.949  |proj g|=       0.12493
## At iterate    34  f =      -26.176  |proj g|=       0.15882
## At iterate    35  f =      -26.719  |proj g|=        0.3604
## At iterate    36  f =       -27.13  |proj g|=       0.39504
## At iterate    37  f =      -27.461  |proj g|=       0.24543
## At iterate    38  f =      -27.722  |proj g|=       0.13591
## At iterate    39  f =      -27.863  |proj g|=      0.043241
## At iterate    40  f =      -27.877  |proj g|=        0.1342
## At iterate    41  f =      -27.877  |proj g|=       0.13417
## At iterate    42  f =      -27.877  |proj g|=       0.10706
## At iterate    43  f =      -27.877  |proj g|=     0.0083219
## At iterate    44  f =      -27.877  |proj g|=     0.0012438
## 
## iterations 44
## function evaluations 54
## segments explored during Cauchy searches 45
## BFGS updates skipped 0
## active bounds at final generalized Cauchy point 0
## norm of the final projected gradient 0.00124382
## final function value -27.8767
## 
## F = -27.8767
## final  value -27.876670 
## converged
```

```
b <- best(design_space, models, DoE)
```

### Algorithm

The above process can be repeated for the desired number of iterations. Here start from the beginning, with another initial design of 20 points followed by 30 iterations of the algorithm.

```
ptm <- proc.time()

design_space <- data.frame(name=c("n","k"), 
                           low=c(100,3), 
                           up=c(500,30)
)

constraints <- data.frame(name=c("beta"), 
                          hyp=c("H1"), 
                          nom=c(0.1),
                          delta=c(0.975))

DoE_num <- 20
dim <- nrow(design_space)
ref <- objective(design_space$up)
  
## Choose initial points
DoE <- data.frame(sobol(DoE_num, dim))
names(DoE) <- design_space$name
for(i in 1:dim){
  DoE[,i] <-  DoE[,i]*(design_space$up[i]-design_space$low[i]) + design_space$low[i]
}
DoE[,1:2] <- round(DoE[,1:2])

## Evaluate at initial points
N <- 100
DoE <- cbind(DoE, t(apply(DoE, 1, calc_rates, h=h, N=N)))
names(DoE)[3:4] <- c("beta", "beta_var")
DoE$N <- N

models <- list()
for(i in 1:nrow(constraints)){
  name <- constraints$name[i]
  models <- append(models, km(~1, design=DoE[1:dim], response=DoE[,as.character(name)], 
                             noise.var=DoE[,paste0(as.character(name), "_var")]))
  names(models)[length(models)] <- as.character(name)
}

b <- best(design_space, models, DoE)

DHs <- NULL

for(i in 1:30){
  opt <- psoptim(rep(NA, 2), exp_improve, lower=design_space$low, upper=design_space$up,
                 N=100, p_set=b, mod=models, design_space=design_space, constraints=constraints,
                 control=list(vectorize = T))
  sol <- opt$par
  sol[1:2] <- round(sol[1:2])
  
  ## track the objective value at each step
  p_set2 <- as.matrix(b[,obj_names])
  current <- dominatedHypervolume(p_set2, ref)
  DHs <- c(DHs, dominatedHypervolume(p_set2, ref))
  
  ## Do the evaluation and add to the design
  y <- calc_rates(sol, h, N=100)
  
  DoE <- rbind(DoE, c(sol, y, 100))
  
  models <- list()
  for(i in 1:nrow(constraints)){
    name <- constraints$name[i]
    models <- append(models, km(~1, design=DoE[1:dim], response=DoE[,as.character(name)], 
                               noise.var=DoE[,paste0(as.character(name), "_var")]))
    names(models)[length(models)] <- as.character(name)
  }
  
  b <- best(design_space, models, DoE)
}

proc.time() - ptm
## 46.759 minutes

## Save the dominated hypervolumes of the approximation sets at each iteration
#saveRDS(DHs, "./data/ex1_single_run_DHs.Rda")

## Save the final DoE table containing all the points evaluated over the whole algorithm
#saveRDS(DoE, "./data/ex1_single_run_DoE.Rda")
```

For comparison, the fixed 50 point Sobol experimental design.

```
## Choose initial points
DoE_num <- 50
DoE <- data.frame(sobol(DoE_num, dim))
names(DoE) <- design_space$name
for(i in 1:dim){
  DoE[,i] <-  DoE[,i]*(design_space$up[i]-design_space$low[i]) + design_space$low[i]
}
DoE[,1:2] <- round(DoE[,1:2])

## Evaluate at initial points
N <- 100
DoE <- cbind(DoE, t(apply(DoE, 1, calc_rates, h=h, N=N)))
names(DoE)[3:4] <- c("beta", "beta_var")
DoE$N <- N

## Discard any solutions which are not probably powered
DoE$pow_lim <- DoE$beta + qnorm(0.975)*sqrt(DoE$beta_var)
DoE <- DoE[DoE$pow_lim <= 0.1,]

## Discard any remaining solutions that are dominated
is_nondom <- function(x, b)
{
    i <- 1
    while(i <= nobj & nrow(b)!= 0 ){
      ## subset b to those non-dominated solutions which are less than or equal to 
      ## x in an objective
      b <- b[b[, obj_names[i] ] <= x[[ obj_names[i] ]],]
      i <- i + 1
    }
    ## If b is now empty, then des is non-dominated
    if(nrow(b)==1 | all(apply(b[,c("f1","f2")], 2, function(x) length(unique(x)) == 1) == TRUE) ) {
      nondom <- TRUE
    } else {
      nondom <- FALSE
    }
    return(nondom)
}
 
DoE <- cbind(DoE, t(apply(DoE, 1, objective)))
names(DoE)[(ncol(DoE)-nobj+1):ncol(DoE)] <- obj_names
DoE <- DoE[apply(DoE, 1, is_nondom, b=DoE), ]

#saveRDS(DoE, "./data/ex1_sobol_DoE.Rda")
```

### Results

Plot the final results:

```
DHs <- readRDS("./data/ex1_single_run_DHs.Rda")
DoE <- readRDS("./data/ex1_single_run_DoE.Rda")
DoE_sob <- readRDS("./data/ex1_sobol_DoE.Rda")

models <- list()
for(i in 1:nrow(constraints)){
  name <- constraints$name[i]
  models <- append(models, km(~1, design=DoE[1:dim], response=DoE[,as.character(name)], 
                             noise.var=DoE[,paste0(as.character(name), "_var")]))
  names(models)[length(models)] <- as.character(name)
}
```

```
## 
## optimisation start
## ------------------
## * estimation method   : MLE 
## * optimisation method : BFGS 
## * analytical gradient : used
## * trend model : ~1
## * covariance model : 
##   - type :  matern5_2 
##   - noise variances :
##  [1] 0.0008272727 0.0002939394 0.0016161616 0.0009888889 0.0003878788
##  [6] 0.0004797980 0.0020797980 0.0018424242 0.0013575758 0.0011424242
## [11] 0.0007434343 0.0011424242 0.0004797980 0.0004797980 0.0021212121
## [16] 0.0022333333 0.0016757576 0.0001979798 0.0019909091 0.0010666667
## [21] 0.0003878788 0.0005696970 0.0016161616 0.0009090909 0.0008272727
## [26] 0.0006575758 0.0009090909 0.0006575758 0.0008272727 0.0009888889
## [31] 0.0005696970 0.0009888889 0.0010666667 0.0009090909 0.0007434343
## [36] 0.0009090909 0.0024757576 0.0006575758 0.0008272727 0.0008272727
## [41] 0.0007434343 0.0004797980 0.0009888889 0.0008272727 0.0009888889
## [46] 0.0010666667 0.0006575758 0.0006575758 0.0010666667 0.0009888889
##   - parameters lower bounds :  1e-10 1e-10 
##   - parameters upper bounds :  750 52 
##   - variance bounds :  0.0004538487 0.1162583 
##   - best initial criterion value(s) :  90.76882 
## 
## N = 3, M = 5 machine precision = 2.22045e-16
## At X0, 0 variables are exactly at the bounds
## At iterate     0  f=      -90.769  |proj g|=      0.22579
## At iterate     1  f =      -91.182  |proj g|=       0.34339
## At iterate     2  f =      -91.195  |proj g|=       0.33398
## At iterate     3  f =        -91.2  |proj g|=       0.32124
## At iterate     4  f =        -91.2  |proj g|=       0.32328
## At iterate     5  f =      -91.201  |proj g|=       0.32419
## At iterate     6  f =       -91.21  |proj g|=        0.3286
## At iterate     7  f =       -91.23  |proj g|=       0.33099
## At iterate     8  f =      -91.282  |proj g|=       0.32625
## At iterate     9  f =      -91.388  |proj g|=       0.30168
## At iterate    10  f =       -91.58  |proj g|=       0.23831
## At iterate    11  f =      -91.935  |proj g|=      0.097642
## At iterate    12  f =      -92.038  |proj g|=      0.026824
## At iterate    13  f =      -92.053  |proj g|=      0.025122
## At iterate    14  f =      -92.056  |proj g|=      0.092167
## At iterate    15  f =      -92.056  |proj g|=      0.023948
## At iterate    16  f =      -92.056  |proj g|=     0.0030599
## At iterate    17  f =      -92.056  |proj g|=     0.0029874
## 
## iterations 17
## function evaluations 22
## segments explored during Cauchy searches 18
## BFGS updates skipped 0
## active bounds at final generalized Cauchy point 0
## norm of the final projected gradient 0.00298744
## final function value -92.0564
## 
## F = -92.0564
## final  value -92.056417 
## converged
```

```
## The final approximation set
b <- best(design_space, models, DoE)
## Extend to include extreme points for plotting
b2 <- b[,1:2]
b2 <- rbind(c(min(b2[,1]), 30), b2, c(500, min(b2[,2])))

## Fixed design comparator
b3 <- DoE_sob[,1:2]
b3 <- rbind(c(min(b3[,1]), 30), b3, c(500, min(b3[,2])))

## Get the GP predictions
mod <- models[[1]]
df <- expand.grid(n = seq(100,500,10), k=3:30)
pred <- predict(mod, newdata=df, type="SK")
df$beta <- pred$mean
df$sd <- pred$sd
```

```
b2$beta <- 1; b3$beta <- 1
## Plot evaluated points With the mean function
ggplot(df, aes(2*n, 3*k, z=beta)) + geom_contour(colour="light blue") + 
  geom_point(data=DoE[1:20,], colour=colours[1], shape=1) +
  geom_point(data=DoE[21:60,], colour=colours[2], shape=4) +
  
  geom_point(data=b2[2:(nrow(b2)-1),], colour=colours[3]) +
  geom_step(data=b2, colour=colours[3], linetype=2) +
  
  geom_point(data=b3[2:(nrow(b3)-1),], colour=colours[5]) +
  geom_step(data=b3, colour=colours[5], linetype=2) +
  
  theme_minimal() + xlab("Number of participants") + ylab("Number of providers")
```

```
## Warning: Removed 10 rows containing missing values (geom_point).
```

```
#ggsave("./paper/figures/ex1_single_run.pdf", height=9, width=14, units="cm")
#ggsave("./paper/figures/ex1_single_run.eps", height=9, width=14, units="cm")
#ggsave("./paper/figures/ex1_single_run.png", height=3, width=5)

## Plot dominated hypervolumes over the search
df2 <- data.frame(it = 1:30, DH=DHs)
ggplot(df2, aes(it, DH)) + geom_point() + geom_line() + 
  theme_minimal() + ylab("Dominated hypervolume") + xlab("Iteration")
```

```
#ggsave("./paper/figures/ex1_traj.pdf", height=9, width=14, units="cm")
#ggsave("./paper/figures/ex1_traj.eps", height=9, width=14, units="cm")
```

For each solution in the approximation set, we can verify whether or not it really is within the type II error rate nominal bound by re-evaluating it using \(N = 50,000\) MC samples.

```
tab <- b[,1:4]
new_eval <- NULL
for(i in 1:nrow(tab)){
  new_eval <- rbind(new_eval, calc_rates(as.numeric(tab[i, 1:2]), h=h, N=50000))
}
tab <- cbind(tab, new_eval)
names(tab)[5:6] <- c("beta_2", "beta_var_2")

#saveRDS(tab, "./data/ex1_large_N.Rda")
```

```
tab <- readRDS("./data/ex1_large_N.Rda")

## Print the results in a table
tab2 <- data.frame(n=tab$n*2, k=tab$k, j=tab$k*2, 
                   beta=apply(tab[,3:4], 1, function(x) paste0(round(x[1], 2), " (", round(sqrt(x[2]), 3), ")")),
                   beta_2=apply(tab[,5:6], 1, function(x) paste0(round(x[1], 3), " (", round(sqrt(x[2]), 3), ")"))
                   )
tab2 <- tab2[order(tab2$k),]
colnames(tab2) <- c("$2n$", "$k$", "$j$", "$\\beta$ (s.e.), $N = 10^2$", "$\\beta$ (s.e.), $N = 50^4$")
tab2
```

```
##    $2n$ $k$ $j$ $\\beta$ (s.e.), $N = 10^2$ $\\beta$ (s.e.), $N = 50^4$
## 30  742   6  12                0.11 (0.031)               0.093 (0.003)
## 24  678   7  14                  0.1 (0.03)               0.081 (0.003)
## 45  626   8  16                0.11 (0.031)               0.087 (0.003)
## 40  604   9  18                0.09 (0.029)               0.077 (0.003)
## 22  564  10  20                0.06 (0.024)               0.081 (0.003)
## 44  546  12  24                0.09 (0.029)               0.084 (0.003)
```

```
# print(xtable(tab2, digits=0), booktabs = T, include.rownames = F, 
#       sanitize.text.function = function(x) {x}, floating = F,
#       file = "./paper/tables/ex1_results.txt")
```

Now plot the results as an animation:

## Example 2

We extend the problem above to an analysis of two binary endpoints, fatigue and disability.

### Simualtion

```
sim_trial2 <- function(x, h)
{
  n <- x[1]; k <- x[2]
  j <- 2*k
  
  r_t <- h[1]; r_d <- h[2]; v_w <- h[3]; cov_res <- h[4]; cov_w <- h[5]
  p0a <- h[6]; p1a <- h[7]; p0b <- h[8]; p1b <- h[9]
  
  ## Find the terms for the linear predictor which correspond to the probabilities
  lp0a <- log(p0a/(1-p0a)); lp1a <- log(p1a/(1-p1a)) - lp0a
  lp0b <- log(p0b/(1-p0b)); lp1b <- log(p1b/(1-p1b)) - lp0b
  
  ## Doctor effects
  v_d <- r_d*v_w/(1-r_d)
  doc <- cbind(seq(1,j), rmvnorm(j, c(0,0), matrix(c(v_d, v_d*cov_res, v_d*cov_res, v_d), ncol=2, byrow = 2)))
  
  ## Therapist effects
  v_t <- (r_t*v_d + r_t*v_w)/(1-r_t)
  ther <- cbind(seq(0,k), rbind(c(0, 0), rmvnorm(k, c(0,0), matrix(c(v_t, v_t*cov_res, v_t*cov_res, v_t), ncol=2, byrow = 2))))
  
  ## 1   | 2    | 3   | 4    | 5    | 6     | 7
  ## arm | p_id |d_id | d_ef | t_id | t_eff | outcome
  
  ## Treatment group
  trt <- c(rep(0, n), rep(1, n))
  ## Patient ID
  p_id <- seq(1, 2*n)
  ## Doctor allocation and effect
  z <- rgamma(j, 1)
  ms <- round(z*2*n/sum(z))
  ms[which.max(ms)] <- ms[which.max(ms)] + 2*n - sum(ms)
  d_id <- sample(rep(doc[,1], ms))
  d_eff_a <- doc[d_id,2]
  d_eff_b <- doc[d_id,3]
  ## Therapist allocation and effect
  z <- rgamma(k, 1)
  ms <- round(z*n/sum(z))
  ms[which.max(ms)] <- ms[which.max(ms)] + n - sum(ms)
  t_id <- c(rep(0, n), sample(rep(ther[2:(k+1),1], ms)))
  t_eff_a <- ther[t_id+1,2]
  t_eff_b <- ther[t_id+1,3]
  ## Residual - now bi-variate
  resid_n <- rmvnorm(2*n, c(0,0), matrix(c(1, cov_w, cov_w, 1), ncol=2, byrow = 2))
  ps <- pnorm(resid_n)
  resid_log  <- qlogis(ps)

  data_a <- cbind(trt,p_id,d_id,d_eff_a,t_id,t_eff_a,resid_log[,1])
  data_b <- cbind(trt,p_id,d_id,d_eff_b,t_id,t_eff_b,resid_log[,2])
  
  ## Outcomes
  data_a <- cbind(data_a, apply(data_a, 1, function(y, lp1, lp0) lp0 + y[1]*lp1 + y[4] + y[6] + y[7], lp1=lp1a, lp0=lp0a))
  data_a <- cbind(data_a, data_a[,8] > 0)
  data_b <- cbind(data_b, apply(data_b, 1, function(y, lp1, lp0) lp0 + y[1]*lp1 + y[4] + y[6] + y[7], lp1=lp1b, lp0=lp0b))
  data_b <- cbind(data_b, data_b[,8] > 0)
    
  ## Analyse the data
  df_a <- as.data.frame(data_a) 
  names(df_a)[9] <- c("y")
  df_b <- as.data.frame(data_b) 
  names(df_b)[9] <- c("y")
  
  result_a <- tryCatch({
    fit1 <- suppressMessages(glmer(y ~ trt + (0 + trt|t_id) + (1|d_id), family = "binomial", data=df_a))
    fit2 <- suppressMessages(glmer(y ~ (0 + trt|t_id) + (1|d_id), family = "binomial", data=df_a))
    p <- anova(fit1, fit2)[2,8]
  }, warning = function(war) {
    ## warning handler picks up where error was generated
    return(1)
  }, error = function(err) {
    ## error handler picks up where error was generated
    print("error")
    return(1)
  }, finally = {

  })
  
  result_b <- tryCatch({
    fit1 <- suppressMessages(glmer(y ~ trt + (0 + trt|t_id) + (1|d_id), family = "binomial", data=df_b))
    fit2 <- suppressMessages(glmer(y ~ (0 + trt|t_id) + (1|d_id), family = "binomial", data=df_b))
    p <- anova(fit1, fit2)[2,8]
  }, warning = function(war) {
    ## warning handler picks up where error was generated
    return(1)
  }, error = function(err) {
    ## error handler picks up where error was generated
    print("error")
    return(1)
  }, finally = {

  })

  return(c(result_a, result_b))
}
```

```
calc_rates2 <- function(x, h, N)
{
  ps <- replicate(N, sim_trial2(x, h))
  y <- ps[1,] > 0.05 | ps[2,] > 0.05
  res <- c(mean(y), var(y)/N)
  
  res
}

## For example,
x <- c(n=181, k=7)
h <- c(r_t=0.05, r_d=0.1, v_w=3.29, cov_res=0.9, cov_w=0.9, p0a=0.1, p1a=0.25, p0b=0.1, p1b=0.25)

ptm <- proc.time()
calc_rates2(x, h, 10)
```

```
## [1] 0.40000000 0.02666667
```

```
proc.time() - ptm
```

```
##    user  system elapsed 
##    7.01    0.00    7.03
```

### Algorithm

```
set.seed(90307)

ptm <- proc.time()

design_space <- data.frame(name=c("n","k"), 
                           low=c(100,3), 
                           up=c(500,30)
)

constraints <- data.frame(name=c("beta"), 
                          hyp=c("H1"), 
                          nom=c(0.1), 
                          delta=c(0.975))

nobj <- 2

obj_names <- NULL
for(i in 1:nobj){
  obj_names <- c(obj_names, paste0("f",i))
}

objective <- function(x)
{
  c(x[1]*2/5, x[2])
}

DoE_num <- 20
dim <- nrow(design_space)
ref <- objective(design_space$up)
  
## Choose initial points
DoE <- data.frame(sobol(DoE_num, dim))
names(DoE) <- design_space$name
for(i in 1:dim){
  DoE[,i] <-  DoE[,i]*(design_space$up[i]-design_space$low[i]) + design_space$low[i]
}
DoE[,1:2] <- round(DoE[,1:2])
```

```
## Evaluate at initial points
N <- 100
h <- c(r_t=0.05, r_d=0.1, v_w=3.29, cov_res=0.9, cov_w=0.9, p0a=0.1, p1a=0.25, p0b=0.1, p1b=0.25)
DoE <- cbind(DoE, t(apply(DoE, 1, calc_rates2, h=h, N=N)))
names(DoE)[3:4] <- c("beta", "beta_var")
DoE$N <- N

models <- list()
for(i in 1:nrow(constraints)){
  name <- constraints$name[i]
  models <- append(models, km(~1, design=DoE[1:dim], response=DoE[,as.character(name)], 
                             noise.var=DoE[,paste0(as.character(name), "_var")]))
  names(models)[length(models)] <- as.character(name)
}

## Get the current set of Pareto solutions
b <- best(design_space, models, DoE)

DHs <- NULL

proc.time() - ptm

for(i in 1:30){
  opt <- psoptim(rep(NA, 2), exp_improve, lower=design_space$low, upper=design_space$up,
                 N=100, p_set=b, models=models, design_space=design_space, constraints=constraints,
                 control=list(vectorize = T))
  sol <- opt$par
  sol[1:2] <- round(sol[1:2])
  
  ## track the objective value at each step
  p_set2 <- as.matrix(b[,obj_names])
  current <- dominatedHypervolume(p_set2, ref)
  DHs <- c(DHs, dominatedHypervolume(p_set2, ref))
  
  ## Do the evaluation and add to the design
  y <- calc_rates2(sol, h, N=100)
  
  DoE <- rbind(DoE, c(sol, y, 100))
  
  models <- list()
  for(i in 1:nrow(constraints)){
    name <- constraints$name[i]
    models <- append(models, km(~1, design=DoE[1:dim], response=DoE[,as.character(name)], 
                             noise.var=DoE[,paste0(as.character(name), "_var")]))
    names(models)[length(models)] <- as.character(name)
  }

  b <- best(design_space, models, DoE)
}

proc.time() - ptm
# 96.33883 minutes

## Save the dominated hypervolumes of the approximation sets at each iteration
#saveRDS(DHs, "./data/ex2_single_run_DHs.Rda")

## Save the final DoE table containing all the points evaluated over the whole algorithm
#saveRDS(DoE, "./data/ex2_single_run_DoE.Rda")
```

### Results

```
DoE <- readRDS("./data/ex2_single_run_DoE.Rda")
DHs <- readRDS("./data/ex2_single_run_DHs.Rda")

models <- list()
for(i in 1:nrow(constraints)){
  name <- constraints$name[i]
  models <- append(models, km(~1, design=DoE[1:dim], response=DoE[,as.character(name)], 
                             noise.var=DoE[,paste0(as.character(name), "_var")]))
  names(models)[length(models)] <- as.character(name)
}
```

```
## 
## optimisation start
## ------------------
## * estimation method   : MLE 
## * optimisation method : BFGS 
## * analytical gradient : used
## * trend model : ~1
## * covariance model : 
##   - type :  matern5_2 
##   - noise variances :
##  [1] 0.0009888889 0.0006575758 0.0025000000 0.0017888889 0.0009090909
##  [6] 0.0014252525 0.0024434343 0.0025090909 0.0016757576 0.0014909091
## [11] 0.0018939394 0.0019434343 0.0003878788 0.0011424242 0.0025242424
## [16] 0.0024606061 0.0023545455 0.0005696970 0.0025242424 0.0012161616
## [21] 0.0003878788 0.0006575758 0.0004797980 0.0003878788 0.0005696970
## [26] 0.0005696970 0.0004797980 0.0004797980 0.0009888889 0.0005696970
## [31] 0.0007434343 0.0008272727 0.0010666667 0.0002939394 0.0023797980
## [36] 0.0004797980 0.0008272727 0.0009888889 0.0008272727 0.0012878788
## [41] 0.0004797980 0.0007434343 0.0006575758 0.0005696970 0.0009090909
## [46] 0.0008272727 0.0007434343 0.0006575758 0.0008272727 0.0008272727
##   - parameters lower bounds :  1e-10 1e-10 
##   - parameters upper bounds :  750 52 
##   - variance bounds :  0.002103837 0.4203008 
##   - best initial criterion value(s) :  87.36736 
## 
## N = 3, M = 5 machine precision = 2.22045e-16
## At X0, 0 variables are exactly at the bounds
## At iterate     0  f=      -87.367  |proj g|=      0.38782
## At iterate     1  f =      -87.612  |proj g|=      0.069986
## At iterate     2  f =      -87.652  |proj g|=      0.096884
## At iterate     3  f =      -87.661  |proj g|=       0.37689
## At iterate     4  f =      -87.662  |proj g|=       0.11787
## At iterate     5  f =      -87.662  |proj g|=       0.11844
## At iterate     6  f =      -87.662  |proj g|=       0.11872
## At iterate     7  f =      -87.662  |proj g|=       0.30775
## At iterate     8  f =      -87.662  |proj g|=       0.37646
## At iterate     9  f =      -87.662  |proj g|=       0.37669
## At iterate    10  f =      -87.663  |proj g|=       0.37706
## At iterate    11  f =      -87.665  |proj g|=       0.37768
## At iterate    12  f =      -87.671  |proj g|=       0.37873
## At iterate    13  f =      -87.685  |proj g|=       0.38047
## At iterate    14  f =       -87.72  |proj g|=       0.38315
## At iterate    15  f =      -87.793  |proj g|=       0.38621
## At iterate    16  f =      -87.883  |proj g|=       0.38664
## At iterate    17  f =      -87.896  |proj g|=       0.38495
## At iterate    18  f =      -87.896  |proj g|=       0.17561
## At iterate    19  f =      -87.896  |proj g|=      0.021638
## At iterate    20  f =      -87.896  |proj g|=      0.027961
## At iterate    21  f =      -87.896  |proj g|=      0.033671
## At iterate    22  f =      -87.896  |proj g|=      0.033706
## At iterate    23  f =      -87.896  |proj g|=      0.033752
## At iterate    24  f =      -87.896  |proj g|=      0.033833
## At iterate    25  f =      -87.896  |proj g|=      0.033956
## At iterate    26  f =      -87.896  |proj g|=      0.034137
## At iterate    27  f =      -87.897  |proj g|=       0.03439
## At iterate    28  f =      -87.898  |proj g|=      0.034703
## At iterate    29  f =      -87.898  |proj g|=       0.03499
## At iterate    30  f =      -87.901  |proj g|=      0.035385
## At iterate    31  f =      -87.962  |proj g|=      0.036985
## At iterate    32  f =      -88.215  |proj g|=       0.37344
## At iterate    33  f =       -88.25  |proj g|=       0.37091
## At iterate    34  f =      -88.255  |proj g|=       0.36994
## At iterate    35  f =      -88.255  |proj g|=      0.060701
## At iterate    36  f =      -88.255  |proj g|=     0.0097717
## At iterate    37  f =      -88.255  |proj g|=     0.0076837
## At iterate    38  f =      -88.255  |proj g|=     0.0032899
## At iterate    39  f =      -88.255  |proj g|=    0.00012693
## 
## iterations 39
## function evaluations 47
## segments explored during Cauchy searches 40
## BFGS updates skipped 0
## active bounds at final generalized Cauchy point 0
## norm of the final projected gradient 0.000126927
## final function value -88.2548
## 
## F = -88.2548
## final  value -88.254825 
## converged
```

```
b <- best(design_space, models, DoE)
b2 <- b[,1:2]
b2 <- rbind(c(min(b2[,1]), 30), b2, c(500, min(b2[,2])))

# Get the GP predictions
mod <- models[[1]]
df <- expand.grid(n = seq(100,500,10), k=3:30)
pred <- predict(mod, newdata=df, type="SK")
df$beta <- pred$mean
df$sd <- pred$sd
```

```
ggplot(df, aes(2*n, 3*k)) + geom_contour(aes(z=beta), colour = "light blue") + 
  geom_point(data=DoE[1:20,], colour=colours[1], shape=1) +
  geom_point(data=DoE[21:50,], colour=colours[2], shape=4) +
  
  geom_point(data=b2[2:(nrow(b2)-1),], colour=colours[3]) +
  geom_step(data=b2, colour=colours[3], linetype=2) +

  theme_minimal() + xlab("Number of participants") + ylab("Number of providers")
```

```
#ggsave("./paper/figures/ex2_single_run.pdf", height=9, width=14, units="cm")
#ggsave("./paper/figures/ex2_single_run.eps", height=9, width=14, units="cm")

## Plot dominated hypervolumes over the search
df2 <- data.frame(it = 1:30, DH=DHs)
ggplot(df2, aes(it, DH)) + geom_point() + geom_line() + 
  theme_minimal() + ylab("Dominated hypervolume") + xlab("Iteration")
```

```
#ggsave("./paper/figures/ex2_traj.pdf", height=9, width=14, units="cm")
#ggsave("./paper/figures/ex2_traj.eps", height=9, width=14, units="cm")
```

```
tab <- b[,1:4]
new_eval <- NULL
for(i in 1:nrow(tab)){
  new_eval <- rbind(new_eval, calc_rates2(as.numeric(tab[i, 1:2]), h=h, N=10000))
}
tab <- cbind(tab, new_eval)
names(tab)[5:6] <- c("beta_2", "beta_var_2")

#saveRDS(tab, "./data/ex2_large_N.Rda")
```

```
## Print the results in a table
tab <- readRDS("./data/ex2_large_N.Rda")

tab2 <- data.frame(n=tab$n*2, k=tab$k, j=tab$k*2, 
                   beta=apply(tab[,3:4], 1, function(x) paste0(round(x[1], 2), " (", round(sqrt(x[2]), 3), ")")),
                   beta_2=apply(tab[,5:6], 1, function(x) paste0(round(x[1], 3), " (", round(sqrt(x[2]), 3), ")"))
                   )
tab2 <- tab2[order(tab2$k),]
colnames(tab2) <- c("$2n$", "$k$", "$j$", "$\\beta$ (s.e.), $N = 10^2$", "$\\beta$ (s.e.), $N = 50^4$")
tab2
```

```
##    $2n$ $k$ $j$ $\\beta$ (s.e.), $N = 10^2$ $\\beta$ (s.e.), $N = 50^4$
## 33  846   9  18                0.12 (0.033)               0.069 (0.003)
## 50  638  10  20                0.09 (0.029)               0.104 (0.003)
## 39  624  12  24                0.09 (0.029)               0.096 (0.003)
```

```
# print(xtable(tab2, digits=0), booktabs = T, include.rownames = F, 
#       sanitize.text.function = function(x) {x}, floating = F,
#       file = "./paper/tables/ex2_results.txt")
```

## Example 3

### Simualtion

```
sim_trial3 <- function(x, h)
{
  n_1 <- x[1]; k <- x[2]; r <- x[3]; j <- x[4]
  n_0 <- round(r*n_1); n_t <- n_0 + n_1
  
  r_t <- h[1]; r_d <- h[2]; v_w <- h[3]; cov_res <- h[4]; cov_w <- h[5]
  p0a <- h[6]; p1a <- h[7]; p0b <- h[8]; p1b <- h[9]
  
  # Find the terms for the linear predictor which correspond to the probabilities
  lp0a <- log(p0a/(1-p0a)); lp1a <- log(p1a/(1-p1a)) - lp0a
  lp0b <- log(p0b/(1-p0b)); lp1b <- log(p1b/(1-p1b)) - lp0b
  
  # Doctor effects
  v_d <- r_d*v_w/(1-r_d)
  doc <- cbind(seq(1,j), rmvnorm(j, c(0,0), matrix(c(v_d, v_d*cov_res, v_d*cov_res, v_d), ncol=2, byrow = 2)))
  
  # Therapist effects
  v_t <- (r_t*v_d + r_t*v_w)/(1-r_t)
  ther <- cbind(seq(0,k), rbind(c(0, 0), rmvnorm(k, c(0,0), matrix(c(v_t, v_t*cov_res, v_t*cov_res, v_t), ncol=2, byrow = 2))))
  
  # 1   | 2    | 3   | 4    | 5    | 6     | 7
  # arm | p_id |d_id | d_ef | t_id | t_eff | outcome
  
  # Treatment group
  trt <- c(rep(0, n_0), rep(1, n_1))
  # Patient ID
  p_id <- seq(1, n_t)
  # Doctor allocation and effect
  d_id <- sample(rep(1:j, ceiling(n_t/j)*j)[1:n_t])
  d_eff_a <- doc[d_id,2]
  d_eff_b <- doc[d_id,3]
  # Therapist allocation and effect
  t_id <- c(rep(0, n_0), rep(1:k, ceiling(n_1/k)*k)[1:n_1])
  t_eff_a <- ther[t_id+1,2]
  t_eff_b <- ther[t_id+1,3]
  # Residual - now bi-variate
  resid_n <- rmvnorm(n_t, c(0,0), matrix(c(v_w, cov_w*v_w, cov_w*v_w, v_w), ncol=2, byrow = 2))
  
  data_a <- cbind(trt,p_id,d_id,d_eff_a,t_id,t_eff_a,resid_n[,1])
  data_b <- cbind(trt,p_id,d_id,d_eff_b,t_id,t_eff_b,resid_n[,2])
  
  var(data_a[,7])
  
  # Outcomes
  data_a <- cbind(data_a, apply(data_a, 1, function(y, lp1, lp0) lp0 + y[1]*lp1 + y[4] + y[6] + y[7], lp1=lp1a, lp0=lp0a))
  data_b <- cbind(data_b, apply(data_b, 1, function(y, lp1, lp0) lp0 + y[1]*lp1 + y[4] + y[6] + y[7], lp1=lp1b, lp0=lp0b))
    
  # Analyse the data
  df_a <- as.data.frame(data_a) 
  names(df_a)[8] <- c("y")
  df_b <- as.data.frame(data_b) 
  names(df_b)[8] <- c("y")
  
  result_a <- tryCatch({
    fit1 <- suppressMessages(lmer(y ~ trt + (0 + trt|t_id) + (1|d_id), REML=F, data=df_a))
    fit2 <- suppressMessages(lmer(y ~ (0 + trt|t_id) + (1|d_id), REML=F, data=df_a))
    p <- anova(fit1, fit2)[2,8]
  }, warning = function(war) {
    # warning handler picks up where error was generated
    #print("warning")
    return(1)
  }, error = function(err) {
    # error handler picks up where error was generated
    print("error")
    return(1)
  }, finally = {

  })
  
  result_b <- tryCatch({
    fit1 <- suppressMessages(lmer(y ~ trt + (0 + trt|t_id) + (1|d_id), REML=F, data=df_b))
    fit2 <- suppressMessages(lmer(y ~ (0 + trt|t_id) + (1|d_id), REML=F, data=df_b))
    p <- anova(fit1, fit2)[2,8]
  }, warning = function(war) {
    # warning handler picks up where error was generated
    #print("warning")
    return(1)
  }, error = function(err) {
    # error handler picks up where error was generated
    print("error")
    return(1)
  }, finally = {

  })

  return(c(result_a, result_b))
}
```

```
calc_rates3 <- function(x, h, N)
{
  alpha <- x[5]
  
  # Alternative hypothesis - one of the endpints is non-null
  ha <- h; ha["p1b"] <- ha["p0b"]
  psa <- replicate(N, sim_trial3(x, ha))
  ya <- psa[1,] > alpha & psa[2,] > alpha
  res_a <- c(mean(ya), var(ya)/N)
  
  # Null hypothesis - both of the endpoints are null
  hn <- h; hn["p1a"] <- hn["p0a"]; hn["p1b"] <- hn["p0b"]
  psn <- replicate(N, sim_trial3(x, hn))
  yn <- psn[1,] < alpha | psn[2,] < alpha
  res_n <- c(mean(yn), var(yn)/N)
  
  c(res_a, res_n)#, mean(psa==10))
}

# For example,
x <- c(n_1=50, k=7, r=1, j=5, a=0.2)
h <- c(r_t=0.05, r_d=0.1, v_w=3.29, cov_res=0.9, cov_w=0.9, p0a=0.1, p1a=0.25, p0b=0.1, p1b=0.25)

ptm <- proc.time()
calc_rates3(x, h, 10)
```

```
## [1] 0.00000000 0.00000000 0.30000000 0.02333333
```

```
proc.time() - ptm
```

```
##    user  system elapsed 
##    2.06    0.00    2.07
```

### Algorithm

```
set.seed(90309)

ptm <- proc.time()

constraints <- data.frame(name=c("beta", "alpha"), 
                          hyp=c("H1", "H0"), 
                          nom=c(0.1, 0.2), 
                          delta=c(0.975,0.975))

design_space <- data.frame(name=c("n_1","k", "r","j","a"), 
                           low=c(20,2,0.5,3, 0.2), 
                           up=c(100,10,1.5,25, 0.01)
                           )

h <- c(r_t=0.05, r_d=0.1, v_w=3.29, cov_res=0.9, cov_w=0.9, p0a=0.1, p1a=0.25, p0b=0.1, p1b=0.25)

dim <- nrow(design_space)
nobj <- 3
obj_names <- NULL
for(i in 1:nobj){
  obj_names <- c(obj_names, paste0("f",i))
}
  
objective <- function(x)
{
  c((x[1]+x[1]*x[3])/5, x[2], x[4])
}

DoE_num <- 50
dim <- nrow(design_space)
  
# Choose initial points
DoE <- data.frame(sobol(DoE_num, dim))
names(DoE) <- design_space$name
for(i in 1:dim){
  DoE[,i] <-  DoE[,i]*(design_space$up[i]-design_space$low[i]) + design_space$low[i]
}
DoE[,c("n_1", "k", "j")] <- round(DoE[,c("n_1", "k", "j")])
```

```
# Evaluate at initial points
N <- 100
DoE <- cbind(DoE, t(apply(DoE, 1, calc_rates3, h=h, N=N)))
names(DoE)[6:9] <- c("beta", "beta_var", "alpha", "alpha_var")
DoE$N <- N

proc.time() - ptm

models <- list()
for(i in 1:nrow(constraints)){
  name <- constraints$name[i]
  models <- append(models, km(~1, design=DoE[1:dim], response=DoE[,as.character(name)], 
                             noise.var=DoE[,paste0(as.character(name), "_var")]))
  names(models)[length(models)] <- as.character(name)
}

# Get the current set of Pareto solutions
b <- best(design_space, models, DoE)

ref <- objective(design_space$up)
DHs <- NULL

for(i in 1:150){
  opt <- psoptim(rep(NA, 5), exp_improve, lower=design_space$low, upper=design_space$up,
                 N=100, p_set=b, models=models, design_space=design_space, constraints=constraints,
                 control=list(vectorize = T))
  sol <- opt$par
  sol[c(1,2,4)] <- round(sol[c(1,2,4)])
  
  # Do the evaluation and add to the design
  y <- calc_rates3(sol, h, N=100)
  
  DoE <- rbind(DoE, c(sol, y, 100))
  
  # track the objective value at each step
  p_set2 <- as.matrix(b[,obj_names])
  current <- dominatedHypervolume(p_set2, ref)
  DHs <- c(DHs, dominatedHypervolume(p_set2, ref))
  
  models <- list()
  for(i in 1:nrow(constraints)){
    name <- constraints$name[i]
    models <- append(models, km(~1, design=DoE[1:dim], response=DoE[,as.character(name)], 
                               noise.var=DoE[,paste0(as.character(name), "_var")]))
    names(models)[length(models)] <- as.character(name)
  }

  b <- best(design_space, models, DoE)
}

proc.time() - ptm
# 156.2025

#saveRDS(DoE, "./data/ex3_single_run_DoE.Rda")
#saveRDS(DHs, "./data/ex3_single_run_DHs.Rda")
```

### Results

```
DoE <- readRDS("./data/ex3_single_run_DoE.Rda")
DHs <- readRDS("./data/ex3_single_run_DHs.Rda")

models <- list()
for(i in 1:nrow(constraints)){
  name <- constraints$name[i]
  models <- append(models, km(~1, design=DoE[1:dim], response=DoE[,as.character(name)], 
                             noise.var=DoE[,paste0(as.character(name), "_var")]))
  names(models)[length(models)] <- as.character(name)
}
```

```
## 
## optimisation start
## ------------------
## * estimation method   : MLE 
## * optimisation method : BFGS 
## * analytical gradient : used
## * trend model : ~1
## * covariance model : 
##   - type :  matern5_2 
##   - noise variances :
##   [1] 0.0009888889 0.0017333333 0.0017888889 0.0025000000 0.0004797980
##   [6] 0.0007434343 0.0022333333 0.0025000000 0.0005696970 0.0009090909
##  [11] 0.0024757576 0.0012161616 0.0016161616 0.0017333333 0.0018939394
##  [16] 0.0020363636 0.0019434343 0.0014909091 0.0010666667 0.0017333333
##  [21] 0.0005696970 0.0009888889 0.0025242424 0.0022666667 0.0012161616
##  [26] 0.0005696970 0.0014252525 0.0019909091 0.0012161616 0.0023272727
##  [31] 0.0025242424 0.0025090909 0.0009090909 0.0017333333 0.0025252525
##  [36] 0.0019434343 0.0014252525 0.0011424242 0.0015545455 0.0010666667
##  [41] 0.0012161616 0.0022333333 0.0007434343 0.0021979798 0.0005696970
##  [46] 0.0008272727 0.0023545455 0.0014909091 0.0009888889 0.0008272727
##  [51] 0.0022979798 0.0009090909 0.0009090909 0.0008272727 0.0014252525
##  [56] 0.0004797980 0.0002939394 0.0012161616 0.0014252525 0.0007434343
##  [61] 0.0005696970 0.0018424242 0.0018424242 0.0008272727 0.0009090909
##  [66] 0.0024242424 0.0008272727 0.0003878788 0.0007434343 0.0007434343
##  [71] 0.0014909091 0.0008272727 0.0017888889 0.0012878788 0.0009888889
##  [76] 0.0008272727 0.0004797980 0.0017333333 0.0011424242 0.0012878788
##  [81] 0.0006575758 0.0009888889 0.0010666667 0.0004797980 0.0006575758
##  [86] 0.0003878788 0.0007434343 0.0013575758 0.0003878788 0.0007434343
##  [91] 0.0012161616 0.0003878788 0.0016161616 0.0004797980 0.0008272727
##  [96] 0.0009090909 0.0009888889 0.0013575758 0.0014909091 0.0012878788
## [101] 0.0012161616 0.0007434343 0.0007434343 0.0008272727 0.0012161616
## [106] 0.0008272727 0.0010666667 0.0009090909 0.0024434343 0.0006575758
## [111] 0.0013575758 0.0012878788 0.0011424242 0.0006575758 0.0005696970
## [116] 0.0011424242 0.0009090909 0.0009090909 0.0002939394 0.0014252525
## [121] 0.0009090909 0.0005696970 0.0011424242 0.0011424242 0.0006575758
## [126] 0.0014252525 0.0005696970 0.0011424242 0.0009888889 0.0009090909
## [131] 0.0012161616 0.0007434343 0.0018939394 0.0008272727 0.0009090909
## [136] 0.0009888889 0.0007434343 0.0006575758 0.0004797980 0.0009090909
## [141] 0.0009888889 0.0005696970 0.0007434343 0.0005696970 0.0012161616
## [146] 0.0009090909 0.0006575758 0.0011424242 0.0009090909 0.0007434343
## [151] 0.0006575758 0.0025252525 0.0015545455 0.0008272727 0.0011424242
## [156] 0.0012878788 0.0008272727 0.0009888889 0.0007434343 0.0008272727
## [161] 0.0009090909 0.0017333333 0.0004797980 0.0009888889 0.0012878788
## [166] 0.0007434343 0.0009090909 0.0013575758 0.0025090909 0.0012878788
## [171] 0.0009888889 0.0019909091 0.0010666667 0.0010666667 0.0012878788
## [176] 0.0009090909 0.0012161616 0.0016757576 0.0012161616 0.0006575758
## [181] 0.0010666667 0.0006575758 0.0010666667 0.0009888889 0.0009888889
## [186] 0.0010666667 0.0017888889 0.0005696970 0.0005696970 0.0012161616
## [191] 0.0006575758 0.0006575758 0.0014252525 0.0003878788 0.0004797980
## [196] 0.0012161616 0.0011424242 0.0006575758 0.0009888889 0.0010666667
##   - parameters lower bounds :  1e-10 1e-10 1e-10 1e-10 1e-10 
##   - parameters upper bounds :  160 16 2 44 0.3750066 
##   - variance bounds :  0.00209863 0.3734565 
##   - best initial criterion value(s) :  321.0864 
## 
## N = 6, M = 5 machine precision = 2.22045e-16
## At X0, 0 variables are exactly at the bounds
## At iterate     0  f=      -321.09  |proj g|=      0.79406
## At iterate     1  f =      -321.56  |proj g|=       0.70715
## At iterate     2  f =      -322.25  |proj g|=       0.67205
## At iterate     3  f =      -322.34  |proj g|=       0.69087
## At iterate     4  f =      -322.34  |proj g|=       0.68807
## At iterate     5  f =      -322.37  |proj g|=       0.68197
## At iterate     6  f =      -322.46  |proj g|=       0.66388
## At iterate     7  f =      -322.66  |proj g|=       0.64962
## At iterate     8  f =      -322.81  |proj g|=       0.64727
## At iterate     9  f =      -322.92  |proj g|=       0.70764
## At iterate    10  f =      -322.96  |proj g|=       0.65307
## At iterate    11  f =      -322.97  |proj g|=       0.66508
## At iterate    12  f =      -322.97  |proj g|=       0.66824
## At iterate    13  f =      -322.97  |proj g|=       0.67344
## At iterate    14  f =      -322.98  |proj g|=       0.68209
## At iterate    15  f =      -322.99  |proj g|=       0.69449
## At iterate    16  f =      -323.03  |proj g|=       0.71388
## At iterate    17  f =      -323.13  |proj g|=       0.73957
## At iterate    18  f =      -323.39  |proj g|=       0.76728
## At iterate    19  f =      -324.07  |proj g|=       0.75455
## At iterate    20  f =      -325.61  |proj g|=       0.52377
## At iterate    21  f =      -326.52  |proj g|=       0.46594
## At iterate    22  f =      -327.41  |proj g|=        1.2985
## At iterate    23  f =      -327.96  |proj g|=       0.47989
## At iterate    24  f =      -328.01  |proj g|=       0.47145
## At iterate    25  f =      -328.01  |proj g|=       0.51189
## At iterate    26  f =      -328.01  |proj g|=       0.51549
## At iterate    27  f =      -328.01  |proj g|=        0.5187
## At iterate    28  f =      -328.01  |proj g|=       0.53611
## At iterate    29  f =      -328.02  |proj g|=       0.55064
## At iterate    30  f =      -328.04  |proj g|=       0.57774
## At iterate    31  f =      -328.14  |proj g|=       0.67149
## At iterate    32  f =      -328.32  |proj g|=       0.66675
## At iterate    33  f =       -328.6  |proj g|=        1.1165
## At iterate    34  f =      -329.13  |proj g|=       0.87901
## At iterate    35  f =      -330.49  |proj g|=       0.38889
## At iterate    36  f =       -332.3  |proj g|=       0.31234
## At iterate    37  f =      -333.51  |proj g|=       0.32595
## At iterate    38  f =      -333.78  |proj g|=       0.33431
## At iterate    39  f =      -333.81  |proj g|=       0.26005
## At iterate    40  f =       -333.9  |proj g|=       0.14165
## At iterate    41  f =      -333.91  |proj g|=       0.32891
## At iterate    42  f =      -333.91  |proj g|=       0.13675
## At iterate    43  f =      -333.91  |proj g|=        0.1366
## At iterate    44  f =      -333.91  |proj g|=       0.32856
## At iterate    45  f =      -333.91  |proj g|=       0.21885
## At iterate    46  f =      -333.91  |proj g|=       0.13686
## At iterate    47  f =      -333.91  |proj g|=       0.13701
## At iterate    48  f =      -333.91  |proj g|=       0.13725
## At iterate    49  f =      -333.91  |proj g|=       0.13758
## At iterate    50  f =      -333.91  |proj g|=       0.13816
## At iterate    51  f =      -333.91  |proj g|=       0.13887
## At iterate    52  f =      -333.91  |proj g|=       0.14023
## At iterate    53  f =      -333.92  |proj g|=       0.14122
## At iterate    54  f =      -333.94  |proj g|=       0.25787
## At iterate    55  f =      -333.96  |proj g|=       0.14196
## At iterate    56  f =      -334.06  |proj g|=       0.25574
## At iterate    57  f =      -334.08  |proj g|=       0.23066
## At iterate    58  f =      -334.17  |proj g|=        0.2934
## At iterate    59  f =      -334.28  |proj g|=       0.36807
## At iterate    60  f =      -334.38  |proj g|=       0.39735
## At iterate    61  f =      -334.53  |proj g|=       0.39554
## At iterate    62  f =      -334.95  |proj g|=       0.31979
## At iterate    63  f =      -335.02  |proj g|=       0.31842
## At iterate    64  f =      -335.12  |proj g|=       0.31535
## At iterate    65  f =      -335.13  |proj g|=       0.31333
## At iterate    66  f =      -335.13  |proj g|=       0.15666
## At iterate    67  f =      -335.13  |proj g|=      0.089586
## At iterate    68  f =      -335.13  |proj g|=       0.10599
## At iterate    69  f =      -335.13  |proj g|=      0.058347
## At iterate    70  f =      -335.13  |proj g|=      0.079518
## At iterate    71  f =      -335.13  |proj g|=      0.037027
## At iterate    72  f =      -335.13  |proj g|=     0.0018933
## 
## iterations 72
## function evaluations 80
## segments explored during Cauchy searches 75
## BFGS updates skipped 0
## active bounds at final generalized Cauchy point 2
## norm of the final projected gradient 0.0018933
## final function value -335.127
## 
## F = -335.127
## final  value -335.126606 
## converged
## 
## optimisation start
## ------------------
## * estimation method   : MLE 
## * optimisation method : BFGS 
## * analytical gradient : used
## * trend model : ~1
## * covariance model : 
##   - type :  matern5_2 
##   - noise variances :
##   [1] 0.0013575758 0.0012161616 0.0017333333 0.0005696970 0.0014909091
##   [6] 0.0019909091 0.0012161616 0.0012878788 0.0020363636 0.0022666667
##  [11] 0.0007434343 0.0015545455 0.0003878788 0.0009090909 0.0017888889
##  [16] 0.0019434343 0.0008272727 0.0011424242 0.0019909091 0.0014252525
##  [21] 0.0017888889 0.0014252525 0.0005696970 0.0009090909 0.0014252525
##  [26] 0.0018939394 0.0005696970 0.0016757576 0.0008272727 0.0001979798
##  [31] 0.0013575758 0.0007434343 0.0016757576 0.0014252525 0.0003878788
##  [36] 0.0020797980 0.0008272727 0.0008272727 0.0020797980 0.0014252525
##  [41] 0.0008272727 0.0002939394 0.0012161616 0.0009090909 0.0015545455
##  [46] 0.0024757576 0.0010666667 0.0001979798 0.0014909091 0.0018424242
##  [51] 0.0001000000 0.0017888889 0.0014909091 0.0010666667 0.0011424242
##  [56] 0.0015545455 0.0009888889 0.0005696970 0.0001979798 0.0013575758
##  [61] 0.0013575758 0.0001979798 0.0000000000 0.0011424242 0.0012878788
##  [66] 0.0001979798 0.0013575758 0.0012878788 0.0016161616 0.0013575758
##  [71] 0.0009090909 0.0014252525 0.0020797980 0.0008272727 0.0012878788
##  [76] 0.0012878788 0.0010666667 0.0001000000 0.0014909091 0.0012161616
##  [81] 0.0016161616 0.0011424242 0.0009090909 0.0016161616 0.0010666667
##  [86] 0.0018424242 0.0016161616 0.0010666667 0.0016161616 0.0017333333
##  [91] 0.0014252525 0.0016757576 0.0018939394 0.0016757576 0.0012878788
##  [96] 0.0017888889 0.0018424242 0.0018939394 0.0001000000 0.0015545455
## [101] 0.0010666667 0.0012878788 0.0014252525 0.0013575758 0.0014252525
## [106] 0.0014252525 0.0020797980 0.0020363636 0.0002939394 0.0017888889
## [111] 0.0010666667 0.0018424242 0.0012878788 0.0015545455 0.0014909091
## [116] 0.0013575758 0.0010666667 0.0010666667 0.0016757576 0.0022979798
## [121] 0.0013575758 0.0000000000 0.0012161616 0.0015545455 0.0012161616
## [126] 0.0011424242 0.0017333333 0.0013575758 0.0014252525 0.0012161616
## [131] 0.0021212121 0.0015545455 0.0001979798 0.0013575758 0.0010666667
## [136] 0.0013575758 0.0009888889 0.0012161616 0.0010666667 0.0014909091
## [141] 0.0011424242 0.0022666667 0.0014252525 0.0014252525 0.0016757576
## [146] 0.0016757576 0.0012878788 0.0016757576 0.0014909091 0.0014909091
## [151] 0.0024434343 0.0001000000 0.0013575758 0.0014252525 0.0014909091
## [156] 0.0014252525 0.0012878788 0.0014909091 0.0016757576 0.0010666667
## [161] 0.0012161616 0.0001000000 0.0014252525 0.0012878788 0.0015545455
## [166] 0.0015545455 0.0012161616 0.0018939394 0.0012878788 0.0001979798
## [171] 0.0015545455 0.0016757576 0.0015545455 0.0014252525 0.0010666667
## [176] 0.0013575758 0.0014909091 0.0001000000 0.0012878788 0.0019909091
## [181] 0.0014252525 0.0013575758 0.0013575758 0.0019909091 0.0012161616
## [186] 0.0011424242 0.0002939394 0.0007434343 0.0012878788 0.0018939394
## [191] 0.0013575758 0.0013575758 0.0011424242 0.0016161616 0.0018939394
## [196] 0.0014909091 0.0016757576 0.0014252525 0.0020797980 0.0010666667
##   - parameters lower bounds :  1e-10 1e-10 1e-10 1e-10 1e-10 
##   - parameters upper bounds :  160 16 2 44 0.3750066 
##   - variance bounds :  0.0003255979 0.06714302 
##   - best initial criterion value(s) :  367.9052 
## 
## N = 6, M = 5 machine precision = 2.22045e-16
## At X0, 0 variables are exactly at the bounds
## At iterate     0  f=      -367.91  |proj g|=      0.54775
## At iterate     1  f =      -368.08  |proj g|=       0.52633
## At iterate     2  f =      -368.11  |proj g|=       0.51189
## At iterate     3  f =      -368.15  |proj g|=       0.52775
## At iterate     4  f =      -368.16  |proj g|=       0.53456
## At iterate     5  f =      -368.17  |proj g|=       0.54029
## At iterate     6  f =       -368.6  |proj g|=       0.60502
## ys=-1.149e-01  -gs= 4.471e-02, BFGS update SKIPPED
## At iterate     7  f =       -368.6  |proj g|=       0.60658
## At iterate     8  f =      -368.61  |proj g|=       0.60581
## At iterate     9  f =      -368.61  |proj g|=       0.60598
## At iterate    10  f =      -368.71  |proj g|=       0.57723
## At iterate    11  f =      -369.07  |proj g|=       0.44736
## At iterate    12  f =      -370.02  |proj g|=       0.34266
## At iterate    13  f =      -370.14  |proj g|=       0.34076
## At iterate    14  f =      -370.22  |proj g|=       0.33776
## At iterate    15  f =      -370.22  |proj g|=       0.33755
## At iterate    16  f =      -370.22  |proj g|=       0.13513
## At iterate    17  f =      -370.22  |proj g|=       0.11473
## At iterate    18  f =      -370.22  |proj g|=       0.11473
## 
## iterations 18
## function evaluations 34
## segments explored during Cauchy searches 21
## BFGS updates skipped 1
## active bounds at final generalized Cauchy point 1
## norm of the final projected gradient 0.114727
## final function value -370.222
## 
## F = -370.222
## final  value -370.222427 
## converged
```

```
# Get the current set of Pareto solutions
b <- best(design_space, models, DoE)

ggplot(b, aes(n_1+n_1*r, k, size=j)) + geom_point() +
  theme_minimal() + xlab("Number of participants") + ylab("Number of therapists") +
  #scale_colour_gradientn(name="No. of doctors", colours=rainbow(2)) +
  scale_size(name="No. of doctors") +
  xlim(c(min(b$n_1 + b$n_1*b$r), max(b$n_1 + b$n_1*b$r))) +  ylim(c(min(b$k), max(b$k)))
```

```
#ggsave("./paper/figures/ex3_single_run.pdf", height=9, width=14, units="cm")
#ggsave("./paper/figures/ex3_single_run.eps", height=9, width=14, units="cm")

## Plot dominated hypervolumes over the search
df2 <- data.frame(it = 1:150, DH=DHs)
ggplot(df2, aes(it, DH)) + geom_point() + geom_line() + 
  theme_minimal() + ylab("Dominated hypervolume") + xlab("Iteration")
```

```
#ggsave("./paper/figures/ex3_traj.pdf", height=9, width=14, units="cm")
#ggsave("./paper/figures/ex3_traj.eps", height=9, width=14, units="cm")
```

```
tab <- b[,1:9]
new_eval <- NULL
for(i in 1:nrow(tab)){
  new_eval <- rbind(new_eval, calc_rates3(as.numeric(tab[i, 1:5]), h=h, N=10000))
}
tab <- cbind(tab, new_eval)
names(tab)[10:13] <- c("beta_2", "beta_var_2", "alpha_2", "alpha_var_2")

#saveRDS(tab, "./data/ex3_large_N.Rda")
```

```
## Print the results in a table
tab <- readRDS("./data/ex3_large_N.Rda")

tab2 <- data.frame(n_1=tab$n_1, n=tab$n_1*(1+tab$r), k=tab$k, j=tab$j, a=tab$a,
                   beta=apply(tab[,6:7], 1, function(x) paste0(round(x[1], 2), " (", round(sqrt(x[2]), 3), ")")),
                   alpha=apply(tab[,8:9], 1, function(x) paste0(round(x[1], 2), " (", round(sqrt(x[2]), 3), ")")),
                   beta_2=apply(tab[,10:11], 1, function(x) paste0(round(x[1], 3), " (", round(sqrt(x[2]), 3), ")")),
                   
                   alpha_2=apply(tab[,12:13], 1, function(x) paste0(round(x[1], 3), " (", round(sqrt(x[2]), 3), ")"))
                   )
tab2 <- tab2[order(tab2$k),]
colnames(tab2) <- c("$n_1$", "$n$", "$k$", "$j$", "$a$",
                    "$\\beta$ (s.e.)", "$\\alpha$ (s.e.)", 
                    "$\\beta$ (s.e.)", "$\\alpha$ (s.e.)")
tab2
```

```
##     $n_1$      $n$ $k$ $j$        $a$ $\\beta$ (s.e.) $\\alpha$ (s.e.)
## 85     94 202.3650   5  15 0.10814082    0.07 (0.026)     0.12 (0.033)
## 82     94 168.5074   6  21 0.12484794    0.11 (0.031)     0.13 (0.034)
## 94     94 213.8370   6  12 0.10582167    0.05 (0.022)     0.21 (0.041)
## 160    84 167.4800   6  23 0.10355810    0.09 (0.029)     0.12 (0.033)
## 55     79 173.6947   7  10 0.12254886    0.17 (0.038)     0.13 (0.034)
## 121    95 202.5539   7   9 0.09232674      0.1 (0.03)     0.16 (0.037)
## 118    75 151.7573   8   9 0.12552875      0.1 (0.03)     0.12 (0.033)
## 159    78 148.9657   8  16 0.12437480    0.08 (0.027)     0.21 (0.041)
## 198    76 142.4842   8  22 0.11591220    0.07 (0.026)     0.17 (0.038)
## 89     80 140.4297   9  17 0.13010345     0.04 (0.02)       0.2 (0.04)
## 196    81 145.7236   9  12 0.13505024    0.14 (0.035)     0.18 (0.039)
## 180    97 189.6800  10   5 0.13633924    0.07 (0.026)     0.27 (0.045)
##     $\\beta$ (s.e.) $\\alpha$ (s.e.)
## 85    0.088 (0.003)     0.17 (0.004)
## 82    0.081 (0.003)    0.179 (0.004)
## 94     0.09 (0.003)    0.157 (0.004)
## 160   0.103 (0.003)    0.147 (0.004)
## 55    0.086 (0.003)    0.171 (0.004)
## 121   0.085 (0.003)    0.134 (0.003)
## 118   0.083 (0.003)    0.175 (0.004)
## 159   0.088 (0.003)    0.171 (0.004)
## 198   0.098 (0.003)    0.156 (0.004)
## 89    0.084 (0.003)    0.176 (0.004)
## 196   0.079 (0.003)    0.181 (0.004)
## 180   0.072 (0.003)    0.178 (0.004)
```

```
#  & & & & & \multicolumn{2}{c}{$N = 10^2$} & \multicolumn{2}{c}{$N = 50^4$} \\
# print(xtable(tab2, digits=c(rep(0,5), 2, rep(0,4))),
#       booktabs = T, include.rownames = F, 
#       sanitize.text.function = function(x) {x}, floating = F,
#       file = "./paper/tables/ex3_results.txt")
```

## Example 4

As suggested by a reviewer, we here add a simple example where the exact result is known. We will look at the power of a two-sample t-test. Although we can calculate power exactly in this case, we replicate the simulation setting by adding some Monet Carlo error to the true power.

### Simualtion

```
calc_rates4 <- function(x, h, N)
{
  n <- x[[1]]; k <- x[[2]]; m <- n/k
  var_t <- h[[2]]; rho <- h[[3]]
  sig_c <- sqrt(var_t*rho + var_t/m - var_t*rho/m)
  pow <- power.t.test(n = k, delta = h[[1]], sd = sig_c)$power
  
  res <- c(1 - rbinom(1, N, pow)/N, pow*(1-pow)/N)
  
  res
}

## For example,
x <- c(n=342, k = 18)
h <- c(mu = 0.3, var_t = 1, rho = 0.05)

ptm <- proc.time()
calc_rates4(x, h, 1000)
```

```
## [1] 0.2020000000 0.0001661564
```

```
proc.time() - ptm
```

```
##    user  system elapsed 
##    0.01    0.00    0.01
```

### Algorithm

The proposed method is stochastic, leading to a different approximation set each time it is run. To understand the variation in the quality of these sets, we run the algorithm 500 times and for each run record the dominated hypervolume trajectories. We will also record the number of solutions in each final set, and the proportion of these which do indeed have sufficient power. In the first run, we will save the full details of all evaluations for illustration later.

```
set.seed(90307)

ptm <- proc.time()

design_space <- data.frame(name=c("n","k"), 
                           low=c(100,10), 
                           up=c(500,100)
)

constraints <- data.frame(name=c("beta"), 
                          hyp=c("H1"), 
                          nom=c(0.1), 
                          delta=c(0.975))

nobj <- 2

obj_names <- NULL
for(i in 1:nobj){
  obj_names <- c(obj_names, paste0("f",i))
}

objective <- function(x)
{
  c(x[1]*2/5, x[2])
}

DoE_num <- 20
dim <- nrow(design_space)
ref <- objective(design_space$up)
```

```
M <- 500
all_res <- NULL
ptm <- proc.time()
DoE_num <- 20
  
for(iter in 1:M){

  ## Choose initial points
  DoE <- data.frame(sobol(DoE_num, dim))
  names(DoE) <- design_space$name
  for(i in 1:dim){
    DoE[,i] <-  DoE[,i]*(design_space$up[i]-design_space$low[i]) + design_space$low[i]
  }
  DoE[,1:2] <- round(DoE[,1:2])
  
  ## Evaluate at initial points
  N <- 100
  h <- c(mu = 0.3, var_t = 1, rho = 0.05)
  DoE <- cbind(DoE, t(apply(DoE, 1, calc_rates4, h=h, N=N)))
  names(DoE)[3:4] <- c("beta", "beta_var")
  DoE$N <- N
  
  models <- list()
  for(i in 1:nrow(constraints)){
    name <- constraints$name[i]
    models <- append(models, km(~1, design=DoE[1:dim], response=DoE[,as.character(name)], 
                               noise.var=DoE[,paste0(as.character(name), "_var")]))
    names(models)[length(models)] <- as.character(name)
  }
  
  ## Get the current set of Pareto solutions
  b <- best(design_space, models, DoE)
  
  DHs <- NULL
  
  proc.time() - ptm
  
  sink("NUL") # suppress output to the console
  
  # For the first run, do 200 iterations and save as an example
  iter <- 50 - DoE_num
  if(M == 1) iter <- 200 - DoE_num
  
  for(i in 1:iter){
    opt <- psoptim(rep(NA, nrow(design_space)), exp_improve, lower=design_space$low, upper=design_space$up,
                   N=100, p_set=b, models=models, design_space=design_space, constraints=constraints,
                   control=list(vectorize = T,max.restart=1, reltol=0.01))
    sol <- opt$par
    sol <- round(sol)
    
    ## track the objective value at each step
    p_set2 <- as.matrix(b[,obj_names])
    current <- dominatedHypervolume(p_set2, ref)
    DHs <- c(DHs, dominatedHypervolume(p_set2, ref))
    
    ## Do the evaluation and add to the design
    y <- calc_rates4(sol, h, N=100)
    
    DoE <- rbind(DoE, c(sol, y, 100))
    
    models <- list()
    for(i in 1:nrow(constraints)){
      name <- constraints$name[i]
      models <- append(models, km(~1, design=DoE[1:dim], response=DoE[,as.character(name)], 
                               noise.var=DoE[,paste0(as.character(name), "_var")]))
      names(models)[length(models)] <- as.character(name)
    }
  
    b <- best(design_space, models, DoE)
    
    if(M == 1) saveRDS(DoE, "./data/ex4_DoE.Rda")
  }
  
  sink(NULL)
  
  res <- c(50, 
           dominatedHypervolume(t(apply(b[,1:2], 1, objective)), ref),
           nrow(b),
           mean(apply(b, 1, function(x) calc_rates4_opt(x[1], x[2], h)) < 100000000))
  
  all_res <- rbind(all_res, res)
}
proc.time() - ptm

df_e <- as.data.frame(all_res)
names(df_e) <- c("i", "DH", "s", "p")

sink(NULL)
#saveRDS(df_e, "./data/ex4_many_runs_10DoE.Rda")
#saveRDS(df_e, "./data/ex4_many_runs_20DoE.Rda")
#saveRDS(df_e, "./data/ex4_many_runs_30DoE.Rda")
```

### Comparison

For comparison, we will compare the method against a fixed design approach. Specifically, we generate a fixed space filling design of 50 solutions 500 times. For each set we estimate power as above (using an artificial MC error) and then find approximation sets based on the first 30, 31, , 50 solutions. We then record trajectories, size of sets, and proportions of powered solutions as above.

First, the exact Pareto set:

```
get_pow4 <- function(n, k, h)
{
  # Get the true power
  m <- n/k
  var_t <- h[[2]]; rho <- h[[3]]
  sig_c <- sqrt(var_t*rho + var_t/m - var_t*rho/m)
  power.t.test(n = k, delta = h[[1]], sd = sig_c)$power
}

calc_rates4_opt <- function(n, k, h)
{
  # An objective function to find optimal n for a given k
  m <- n/k
  var_t <- h[[2]]; rho <- h[[3]]
  sig_c <- sqrt(var_t*rho + var_t/m - var_t*rho/m)
  pow <- power.t.test(n = k, delta = h[[1]], sd = sig_c)$power

  n + 100000000*(pow < 0.9)
}

# For a set of ks, find optimal n
ks <- 10:100
ns <- sapply(ks, function(x) optim(200, calc_rates4_opt, method = "Brent", lower = 10, upper = 1000,
                                   k = x, h = h)$par)
ps <- data.frame(n = ns, k = ks)
ps <- ps[ps$n > 11 & ps$n <= 500,]
```

Now, we get approximation sets using the fixed design approach.

```
set.seed(90307)

# The fixed design approach
ref <- objective(design_space$up)

M <- 500
all_res <- NULL
  
for(iter in 1:M){
  ## Choose initial points
  DoE <- data.frame(sobol(1000, dim))
  names(DoE) <- design_space$name
  for(i in 1:dim){
    DoE[,i] <-  DoE[,i]*(design_space$up[i]-design_space$low[i]) + design_space$low[i]
  }
  DoE[,1:2] <- round(DoE[,1:2])
  
  N <- 100
  h <- c(mu = 0.3, var_t = 1, rho = 0.05)
  DoE <- cbind(DoE, t(apply(DoE, 1, calc_rates4, h=h, N=N)))
  names(DoE)[3:4] <- c("beta", "beta_var")
  DoE$N <- N
  DoE$ci <- DoE$beta + qnorm(0.975)*sqrt(DoE$beta_var)
  
  res <- NULL
  for(j in seq(50,1000,50)){
    sub <- DoE[1:j,]
    fb <- paretoFilter(as.matrix(sub[sub$ci < 0.1, 1:2]))
    # save first run as an example
    if(M == 1 & j == 50){
      saveRDS(fb, "./data/ex4_fb.Rda")
      saveRDS(sub, "./data/ex4_DoE_fb.Rda")
    }
    powered <- mean(apply(fb, 1, function(x) calc_rates4_opt(x[1], x[2], h)) < 100000000)
    dh <- dominatedHypervolume(t(apply(fb, 1, objective)), ref)
    res <- rbind(res, c(j, dh, nrow(fb), powered))
  }
  res <- cbind(res, iter)
  all_res <- rbind(all_res, res)
}

df_f <- as.data.frame(all_res)
# i = internal iteartion count
# DH = dominated hypervolume
# s = size of approximation set
# p = proportion of approximation set which is actually powered
names(df_f) <- c("i", "DH", "s", "p", "r")

#saveRDS(df_f, "./data/ex4_fixed.Rda")
```

```
df_f <- readRDS("./data/ex4_fixed.Rda")
df_f <- unique(df_f[df_f$i %in% c(50, 200, 400, 600, 800, 1000), -5])

# Compare with EGO method

df <- rbind(readRDS("./data/ex4_many_runs1.Rda"),
            readRDS("./data/ex4_many_runs2.Rda"),
            readRDS("./data/ex4_many_runs3.Rda"))

df$t <- "EGO"; df_f$t <- "fix"

df$i <- 10001;
df2 <- rbind(df, df_f)
df2$i <- factor(df2$i, labels = c("50", "200", "400", "600", "800", "1000", "EGO"))

# Get Dh of the actual Pareto set
dh_ps <- dominatedHypervolume(matrix(c(objective(ps)$n, objective(ps)$k), ncol=2), ref)

ggplot(df2, aes(i, DH, fill=t)) + geom_boxplot() +
  theme_minimal() +
  ylab("Dominated area") + xlab("Number of evaluations") +
  #theme(axis.text.y=element_blank(),
   #   axis.title.y=element_blank()) +
  scale_fill_manual(name="Method", values=colours, labels = c("EGO", "Fixed")) +
  geom_hline(yintercept = dh_ps, linetype = 2)
```

```
ggplot(df2, aes(i, s, fill=t)) + geom_boxplot() +
  theme_minimal() +
  ylab("Size of appoximation set") +  xlab("Number of evaluations") +
  #theme(axis.text.y=element_blank(),
   #   axis.title.y=element_blank()) +
  scale_fill_manual(name="Method", values=colours, labels = c("EGO", "Fixed"))
```

```
df3 <- data.frame(i = unique(df2$i), t=c("EGO", rep("Fixed", 6)))
df3$pr <- sapply(df3$i, function(x) mean(df2[df2$i == x,"p"] < 1))
df3
```

```
##      i     t         pr
## 1  EGO   EGO 0.03992016
## 2   50 Fixed 0.02955665
## 3  200 Fixed 0.03822938
## 4  400 Fixed 0.10303030
## 5  600 Fixed 0.19354839
## 6  800 Fixed 0.29898990
## 7 1000 Fixed 0.36947791
```

```
#ggsave("./paper/figures/ex4_many_runs.pdf", height=9, width=14, units="cm")
#ggsave("./paper/figures/ex4_many_runs.eps", height=9, width=14, units="cm", device = cairo_ps())
#ggsave("./paper/figures/ex4_many_runs.png", height=3, width=5)
```

Plot example approximation sets from both methods for one of the runs:

```
# Example EGO run with 50 evals
DoE <- readRDS("./data/ex4_DoE.Rda")[1:50,]
# Example EGO run with 200 evals
DoE2 <- readRDS("./data/ex4_DoE.Rda")
# Example fixed design run approximation set
fb <- readRDS("./data/ex4_fb.Rda")

models <- list()
  for(i in 1:nrow(constraints)){
    name <- constraints$name[i]
    models <- append(models, km(~1, design=DoE[1:dim], response=DoE[,as.character(name)], 
                               noise.var=DoE[,paste0(as.character(name), "_var")]))
    names(models)[length(models)] <- as.character(name)
  }
```

```
## 
## optimisation start
## ------------------
## * estimation method   : MLE 
## * optimisation method : BFGS 
## * analytical gradient : used
## * trend model : ~1
## * covariance model : 
##   - type :  matern5_2 
##   - noise variances :
##  [1] 0.0008291902 0.0007833989 0.0014831188 0.0012929950 0.0001668676
##  [6] 0.0014349834 0.0020773293 0.0020061733 0.0003420267 0.0014897443
## [11] 0.0009324094 0.0018158713 0.0002593609 0.0007624720 0.0022805836
## [16] 0.0022441086 0.0004259688 0.0007323349 0.0011286385 0.0018601207
## [21] 0.0004910885 0.0005413749 0.0005486029 0.0004296168 0.0005888914
## [26] 0.0006501156 0.0005761437 0.0006487959 0.0006515634 0.0006062908
## [31] 0.0006402357 0.0006672161 0.0006589630 0.0006435892 0.0007086031
## [36] 0.0006760435 0.0006621190 0.0006769299 0.0007114007 0.0006407195
## [41] 0.0007101615 0.0007123931 0.0007153902 0.0007393017 0.0007595897
## [46] 0.0007334315 0.0007131553 0.0006766392 0.0007624941 0.0007522854
##   - parameters lower bounds :  1e-10 1e-10 
##   - parameters upper bounds :  700 162 
##   - variance bounds :  0.0004524151 0.1192159 
##   - best initial criterion value(s) :  96.40672 
## 
## N = 3, M = 5 machine precision = 2.22045e-16
## At X0, 0 variables are exactly at the bounds
## At iterate     0  f=      -96.407  |proj g|=      0.11262
## At iterate     1  f =      -96.657  |proj g|=      0.027619
## At iterate     2  f =      -96.756  |proj g|=      0.023608
## At iterate     3  f =      -96.781  |proj g|=       0.11018
## At iterate     4  f =      -96.799  |proj g|=      0.019674
## At iterate     5  f =        -96.8  |proj g|=       0.01907
## At iterate     6  f =        -96.8  |proj g|=      0.053363
## At iterate     7  f =        -96.8  |proj g|=       0.01897
## 
## iterations 7
## function evaluations 10
## segments explored during Cauchy searches 8
## BFGS updates skipped 0
## active bounds at final generalized Cauchy point 0
## norm of the final projected gradient 0.0189696
## final function value -96.7999
## 
## F = -96.7999
## final  value -96.799855 
## converged
```

```
## The final approximation set after 50 - 20 iterations
b <- best(design_space, models, DoE)
## Extend to include extreme points for plotting
b2 <- b[,1:2]
b2 <- rbind(c(min(b2[,1]), 100), b2, c(500, min(b2[,2])))

## Fixed design comparator
b3 <- as.data.frame(fb)
b3 <- rbind(c(min(b3[,1]), 100), b3, c(500, min(b3[,2])))

## Actual Pareto set
b4 <- as.data.frame(ps)
b4 <- rbind(c(min(b4[,1]), 100), b4, c(500, min(b4[,2])))

## Get the GP predictions
mod <- models[[1]]
df <- expand.grid(n = seq(100,500,10), k=10:100)
pred <- predict(mod, newdata=df, type="SK")
df$beta <- pred$mean
df$sd <- pred$sd

models <- list()
  for(i in 1:nrow(constraints)){
    name <- constraints$name[i]
    models <- append(models, km(~1, design=DoE2[1:dim], response=DoE2[,as.character(name)], 
                               noise.var=DoE2[,paste0(as.character(name), "_var")]))
    names(models)[length(models)] <- as.character(name)
  }
```

```
## 
## optimisation start
## ------------------
## * estimation method   : MLE 
## * optimisation method : BFGS 
## * analytical gradient : used
## * trend model : ~1
## * covariance model : 
##   - type :  matern5_2 
##   - noise variances :
##   [1] 0.0008291902 0.0007833989 0.0014831188 0.0012929950 0.0001668676
##   [6] 0.0014349834 0.0020773293 0.0020061733 0.0003420267 0.0014897443
##  [11] 0.0009324094 0.0018158713 0.0002593609 0.0007624720 0.0022805836
##  [16] 0.0022441086 0.0004259688 0.0007323349 0.0011286385 0.0018601207
##  [21] 0.0004910885 0.0005413749 0.0005486029 0.0004296168 0.0005888914
##  [26] 0.0006501156 0.0005761437 0.0006487959 0.0006515634 0.0006062908
##  [31] 0.0006402357 0.0006672161 0.0006589630 0.0006435892 0.0007086031
##  [36] 0.0006760435 0.0006621190 0.0006769299 0.0007114007 0.0006407195
##  [41] 0.0007101615 0.0007123931 0.0007153902 0.0007393017 0.0007595897
##  [46] 0.0007334315 0.0007131553 0.0006766392 0.0007624941 0.0007522854
##  [51] 0.0007991502 0.0007541676 0.0007745185 0.0006750342 0.0007723474
##  [56] 0.0007389609 0.0021961173 0.0007399339 0.0007273931 0.0008448333
##  [61] 0.0008074065 0.0008103162 0.0007328821 0.0007917856 0.0008050076
##  [66] 0.0008268326 0.0008257112 0.0007891082 0.0007635282 0.0007043391
##  [71] 0.0007454681 0.0007791768 0.0009183087 0.0007270941 0.0007627426
##  [76] 0.0023679392 0.0007269902 0.0008448333 0.0008101741 0.0008058360
##  [81] 0.0007915769 0.0008047333 0.0007901615 0.0007516956 0.0007920910
##  [86] 0.0008848310 0.0008786308 0.0008017627 0.0008479394 0.0008520322
##  [91] 0.0007778839 0.0008286400 0.0007868029 0.0007159692 0.0008330482
##  [96] 0.0008434236 0.0008479394 0.0008448333 0.0008093869 0.0007655790
## [101] 0.0007505342 0.0008417423 0.0008010029 0.0009183087 0.0008786308
## [106] 0.0008135926 0.0008786308 0.0007789761 0.0008417423 0.0007626062
## [111] 0.0007623496 0.0008347087 0.0008043365 0.0008199365 0.0008410958
## [116] 0.0008043365 0.0008386662 0.0008417423 0.0008212905 0.0007504170
## [121] 0.0008786308 0.0008448333 0.0008043365 0.0008274246 0.0008179838
## [126] 0.0008448333 0.0008448333 0.0007505092 0.0008045925 0.0008417423
## [131] 0.0008156679 0.0008410958 0.0008356051 0.0008786308 0.0008755526
## [136] 0.0008331272 0.0007623496 0.0008012818 0.0008410958 0.0008386662
## [141] 0.0007563418 0.0007743409 0.0007623107 0.0007965893 0.0008271676
## [146] 0.0008479394 0.0007988283 0.0008104918 0.0008383850 0.0008381518
## [151] 0.0007834451 0.0008480960 0.0007683232 0.0008410958 0.0007623107
## [156] 0.0008135926 0.0008288953 0.0008118890 0.0007564734 0.0008330482
## [161] 0.0008167087 0.0008139758 0.0008167087 0.0008135926 0.0008367756
## [166] 0.0007866066 0.0007623107 0.0023598695 0.0006880793 0.0007750924
## [171] 0.0007765971 0.0007869686 0.0007869686 0.0008003197 0.0007862338
## [176] 0.0008058720 0.0007819768 0.0007882516 0.0007765971 0.0007869686
## [181] 0.0007757485 0.0008298996 0.0007703725 0.0008341692 0.0007477646
## [186] 0.0008376975 0.0008298996 0.0007812783 0.0008298996 0.0007703725
## [191] 0.0008298996 0.0008248741 0.0007651443 0.0008367756 0.0007641943
## [196] 0.0024639570 0.0008376975 0.0007757485 0.0007232916 0.0007686625
##   - parameters lower bounds :  1e-10 1e-10 
##   - parameters upper bounds :  788 180 
##   - variance bounds :  0.0002060141 0.07422497 
##   - best initial criterion value(s) :  392.2428 
## 
## N = 3, M = 5 machine precision = 2.22045e-16
## At X0, 0 variables are exactly at the bounds
## At iterate     0  f=      -392.24  |proj g|=     0.067787
## At iterate     1  f =       -398.3  |proj g|=      0.074019
## At iterate     2  f =       -399.3  |proj g|=       0.04248
## At iterate     3  f =      -399.41  |proj g|=      0.035318
## At iterate     4  f =      -399.42  |proj g|=       0.04244
## At iterate     5  f =      -399.42  |proj g|=      0.033129
## At iterate     6  f =      -399.42  |proj g|=      0.030121
## At iterate     7  f =      -399.42  |proj g|=      0.030068
## 
## iterations 7
## function evaluations 12
## segments explored during Cauchy searches 9
## BFGS updates skipped 0
## active bounds at final generalized Cauchy point 0
## norm of the final projected gradient 0.0300682
## final function value -399.422
## 
## F = -399.422
## final  value -399.421573 
## converged
```

```
## The final approximation set after 200 - 20 iterations
b5 <- best(design_space, models, DoE2)
## Extend to include extreme points for plotting
b5 <- b5[,1:2]
b5 <- rbind(c(min(b5[,1]), 100), b5, c(500, min(b5[,2])))

b2$beta <- 1; b3$beta <- 1; b4$beta <- 1; b5$beta <- 1
## Plot evaluated points With the mean function
ggplot(df, aes(2*n, 2*k, z=beta)) + geom_contour(colour="lightblue") + 
  # Initial EGO points
  geom_point(data=DoE[1:20,], shape=1) +
  # Subsequent EGO evals
  geom_point(data=DoE[21:nrow(DoE),], shape=4) +
  # EGO approximation set (50 evals)
  geom_point(data=b2[2:(nrow(b2)-1),], aes(colour="EGO50")) +
  geom_step(data=b2, aes(colour="EGO50"), linetype=2) +
  # Fixed design approximation set
  geom_point(data=b3[2:(nrow(b3)-1),], aes(colour="FD")) +
  geom_step(data=b3, aes(colour="FD"), linetype=2) +
  # EGO approximation set (200 evals)
  geom_point(data=b5[2:(nrow(b5)-1),], aes(colour="EGO200")) +
  geom_step(data=b5, aes(colour="EGO200"), linetype=2) +
  # True Pareto set
  geom_step(data=b4, aes(colour="PS"), linetype=2) +

  scale_color_manual(name = "",
                        breaks = c("EGO50", "FD", "EGO200", "PS"),
                        values = c("EGO50" = colours[2],
                                   "FD" = colours[3],
                                   "EGO200" = colours[1],
                                   "PS" = colours[4]),
                     labels = c("EGO (50)", "Fixed design", "EGO (200)", "Pareto set")) +
  
  theme_minimal() + xlab("Number of participants") + ylab("Number of clusters") +
  xlim(c(500, 1000)) + ylim(c(45,200))
```

```
## Warning: Removed 1703 rows containing non-finite values (stat_contour).
```

```
## Warning: Removed 10 rows containing missing values (geom_point).
```

```
#ggsave("./paper/figures/ex4_single_run.pdf", height=9, width=17, units="cm")
#ggsave("./paper/figures/ex4_single_run.eps", height=9, width=17, units="cm")
#ggsave("./paper/figures/ex4_single_run.png", height=3, width=5)

# In this example the dominated areas are:
dominatedHypervolume(matrix(c(objective(b2)$n, objective(b2)$k), ncol=2), ref)
```

```
## [1] 4689.6
```

```
dominatedHypervolume(matrix(c(objective(b5)$n, objective(b5)$k), ncol=2), ref)
```

```
## [1] 5332.4
```

```
dominatedHypervolume(matrix(c(objective(b3)$n, objective(b3)$k), ncol=2), ref)
```

```
## [1] 2750.4
```

```
dominatedHypervolume(matrix(c(objective(ps)$n, objective(ps)$k), ncol=2), ref)
```

```
## [1] 6121.47
```

Print the individual EGO (50 evaluations) solutions for a table:

```
DoE <- readRDS("./data/ex4_DoE.Rda")[1:50,]

models <- list()
  for(i in 1:nrow(constraints)){
    name <- constraints$name[i]
    models <- append(models, km(~1, design=DoE[1:dim], response=DoE[,as.character(name)], 
                               noise.var=DoE[,paste0(as.character(name), "_var")]))
    names(models)[length(models)] <- as.character(name)
  }
```

```
## 
## optimisation start
## ------------------
## * estimation method   : MLE 
## * optimisation method : BFGS 
## * analytical gradient : used
## * trend model : ~1
## * covariance model : 
##   - type :  matern5_2 
##   - noise variances :
##  [1] 0.0008291902 0.0007833989 0.0014831188 0.0012929950 0.0001668676
##  [6] 0.0014349834 0.0020773293 0.0020061733 0.0003420267 0.0014897443
## [11] 0.0009324094 0.0018158713 0.0002593609 0.0007624720 0.0022805836
## [16] 0.0022441086 0.0004259688 0.0007323349 0.0011286385 0.0018601207
## [21] 0.0004910885 0.0005413749 0.0005486029 0.0004296168 0.0005888914
## [26] 0.0006501156 0.0005761437 0.0006487959 0.0006515634 0.0006062908
## [31] 0.0006402357 0.0006672161 0.0006589630 0.0006435892 0.0007086031
## [36] 0.0006760435 0.0006621190 0.0006769299 0.0007114007 0.0006407195
## [41] 0.0007101615 0.0007123931 0.0007153902 0.0007393017 0.0007595897
## [46] 0.0007334315 0.0007131553 0.0006766392 0.0007624941 0.0007522854
##   - parameters lower bounds :  1e-10 1e-10 
##   - parameters upper bounds :  700 162 
##   - variance bounds :  0.0004524151 0.1192159 
##   - best initial criterion value(s) :  97.11754 
## 
## N = 3, M = 5 machine precision = 2.22045e-16
## At X0, 0 variables are exactly at the bounds
## At iterate     0  f=      -97.118  |proj g|=      0.10857
## At iterate     1  f =       -97.22  |proj g|=      0.014129
## At iterate     2  f =      -97.229  |proj g|=      0.013453
## At iterate     3  f =       -97.23  |proj g|=       0.10571
## At iterate     4  f =       -97.23  |proj g|=        0.0131
## At iterate     5  f =       -97.23  |proj g|=     0.0051589
## 
## iterations 5
## function evaluations 8
## segments explored during Cauchy searches 6
## BFGS updates skipped 0
## active bounds at final generalized Cauchy point 0
## norm of the final projected gradient 0.00515893
## final function value -97.2304
## 
## F = -97.2304
## final  value -97.230406 
## converged
```

```
## The final approximation set after 50 - 20 iterations
b <- best(design_space, models, DoE)
tab <- DoE[row.names(b),]

## Print the results in a table
tab2 <- data.frame(n=tab$n*2, k=2*tab$k,
                   beta_est=apply(tab[,3:4], 1, function(x) paste0(round(x[1], 2), " (", round(sqrt(x[2]), 3), ")")))

tab2$beta <- round(1-apply(tab2, 1, function(x) get_pow4(as.numeric(x[1])/2, as.numeric(x[2])/2, h)), 3)

tab2 <- tab2[order(tab2$k),]
colnames(tab2) <- c("$2n$", "$2k$", "$\\hat{\\beta}$ (s.e.)", "$\\beta$")
tab2
```

```
##    $2n$ $2k$ $\\hat{\\beta}$ (s.e.) $\\beta$
## 48  834   68           0.06 (0.026)    0.073
## 26  806   74           0.07 (0.025)    0.070
## 36  766   78           0.11 (0.026)    0.073
## 29  750   84            0.1 (0.026)    0.070
## 33  708   94           0.06 (0.026)    0.071
## 14  650   98           0.13 (0.028)    0.083
## 49  632  106           0.07 (0.028)    0.083
## 45  612  118           0.07 (0.028)    0.083
## 44  596  136           0.07 (0.027)    0.080
## 46  588  146           0.08 (0.027)    0.080
```

And similarly, for the fixed design approximation set:

```
DoE <- readRDS("./data/ex4_DoE_fb.Rda")

tab <- DoE[row.names(fb),]

## Print the results in a table
tab2 <- data.frame(n=tab$n*2, k=2*tab$k,
                   beta_est=apply(tab[,3:4], 1, function(x) paste0(round(x[1], 2), " (", round(sqrt(x[2]), 3), ")")))

tab2$beta <- round(1-apply(tab2, 1, function(x) get_pow4(as.numeric(x[1])/2, as.numeric(x[2])/2, h)), 3)

tab2 <- tab2[order(tab2$k),]
colnames(tab2) <- c("$2n$", "$2k$", "$\\hat{\\beta}$ (s.e.)", "$\\beta$")
tab2
```

```
##    $2n$ $2k$ $\\hat{\\beta}$ (s.e.) $\\beta$
## 42  988   80           0.04 (0.019)    0.039
## 26  924   94           0.05 (0.019)    0.036
## 50  862  108           0.05 (0.019)    0.036
## 41  788  124            0.03 (0.02)    0.040
## 25  724  138           0.02 (0.021)    0.047
## 49  662  152           0.05 (0.023)    0.056
## 33  638  158           0.05 (0.024)    0.061
```

## Figures

```
# Plot an example optimisation problem, minimising clusters and patients, with
# a true Pareto front and an approximation front, with the dominated hypervolume
# shaded in.

pf <- data.frame(f1 = c(472, 472, 473, 474, 476, 478, 481, 483, 486, 492, 498,
           508, 518, 528, 536, 548, 560, 573, 595, 624, 658, 706, 782, 1150, 1200),
           f2 = c(30:7, 7))
pf$t <- "p"

as <- data.frame(f2=c(30, 24,20,12,10, 10),
                 f1=c(589, 589, 705, 810, 982, 1200))
as$t <- "a"

df <- rbind(pf, as)

pg <- data.frame(f1 = c(1200, 982,982, 810, 810, 705, 705, 589, 589, 1200),
                 f2 = c(10, 10, 12, 12, 20, 20, 24, 24, 30, 30))

ggplot(df, aes(f1, f2)) + geom_polygon(data=pg, alpha=0.1) + #fill="grey90") + 
  geom_step(aes(colour=t)) + 
  geom_point(data=df[!(df$f1==1200 | df$f2==30),], aes(colour=t)) +
  theme_minimal() + xlab("Number of participants") + ylab("Number of clusters") +
  scale_colour_manual(name= "",
                      values=c(colours[3], colours[4]), 
                      labels=c("Approximation set", "Pareto set")) #+
```

```
  #geom_point(data=data.frame(f1=1200, f2=30), shape=4, size=3, stroke=2) +
  #annotate("text", x = 900, y = 23, label = "H(A)")
  
# ggsave("./paper/figures/fake_pareto.pdf", width= 5, height = 3)
# ggsave("./paper/figures/fake_pareto.eps", width= 5, height = 3, device=cairo_ps)
# ggsave("./paper/figures/fake_pareto.png", width= 4, height = 2)
```

Plot of a GP constraint, annotating some expected improvemets at a couple of points

```
get_power <- function(n)
{
  return(power.t.test(n=n, delta=0.3)$power)
}

df <- data.frame(n = c(10, 110, 260, 290))
df$f<- apply(df, 1, get_power)


model <- km(design = df[,1, drop=FALSE], response = df[,2])
```

```
## 
## optimisation start
## ------------------
## * estimation method   : MLE 
## * optimisation method : BFGS 
## * analytical gradient : used
## * trend model : ~1
## * covariance model : 
##   - type :  matern5_2 
##   - nugget : NO
##   - parameters lower bounds :  1e-10 
##   - parameters upper bounds :  560 
##   - best initial criterion value(s) :  0.3434455 
## 
## N = 1, M = 5 machine precision = 2.22045e-16
## At X0, 0 variables are exactly at the bounds
## At iterate     0  f=     -0.34345  |proj g|=    5.528e-05
## At iterate     1  f =     -0.34345  |proj g|=    5.5279e-05
## At iterate     2  f =     -0.34355  |proj g|=    7.7445e-07
## At iterate     3  f =     -0.34355  |proj g|=    1.1035e-08
## At iterate     4  f =     -0.34355  |proj g|=    2.2416e-12
## 
## iterations 4
## function evaluations 5
## segments explored during Cauchy searches 4
## BFGS updates skipped 0
## active bounds at final generalized Cauchy point 0
## norm of the final projected gradient 2.24161e-12
## final function value -0.343552
## 
## F = -0.343552
## final  value -0.343552 
## converged
```

```
x <- data.frame(n=seq(100, 300))

p <- predict.km(model, newdata=x, type="SK")

x$f <- p$mean
x$sd <- p$sd
x$min <- x$f - 1.96*x$sd
x$max <- x$f + 1.96*x$sd

get_p <- function(input)
{
  return(1-pnorm(0.8, input[2], input[3]))
}

probs <- cbind(x, V1=apply(x, 1, get_p))
probs$EFI <- (260-probs$n)*probs$V1/450 + 0.5
probs$EFI <- ifelse(probs$EFI < 0.5 , 0.5, probs$EFI)

# Optimal expected feasible improvement is at 190, with
# m = 0.8388252, sd = 3.464424e-02

get_n <- function(y, m, s, n)
{
  n + 1.2*dnorm(y, m, s)
}

m <- 0.8388252
s <- 3.464424e-02

x6 <- data.frame(f=c(seq(0,m,0.001), m))
x6$n <- apply(x6, 1, get_n, m=m, s=s, n=190)
x6 <- subset(x6, f>0.5)

x7 <- data.frame(f=seq(m,1,0.001))
x7$n <- apply(x7, 1, get_n, m=m, s=s, n=190)

# For paper
ggplot(x6, aes(n, f)) + geom_line(colour="#009E73", linetype=2) + geom_line(data=x7, colour="#009E73", linetype=2) + 
  geom_line(data=x, colour="#D55E00") + geom_ribbon(data=x, aes(ymin=min, ymax=max), alpha=0.1) + 
  #geom_line(data=x4, colour="red") + geom_line(data=x5, colour="red") +
  geom_point(data=subset(df, n>100)) +
  scale_y_continuous(limits = c(0.5, 1)) +
  geom_line(data=probs, aes(n, EFI), colour="#CC79A7", linetype=3) +
  theme_minimal() + ylab("Power") + xlab("Sample size")
```

```
#ggsave("./paper/figures/GP_example.pdf", width= 5, height = 3.3)
#ggsave("./paper/figures/GP_example.eps", width= 5, height = 3.3, device=cairo_ps)
```
